# Attenuated strain of CVB3 with a mutation in the CAR-interacting region protects against both myocarditis and pancreatitis

**DOI:** 10.1038/s41598-021-90434-w

**Published:** 2021-06-14

**Authors:** Ninaad Lasrado, Arunakumar Gangaplara, Chandirasegaran Massilamany, Rajkumar Arumugam, Allison Shelbourn, Mahima T. Rasquinha, Rakesh H. Basavalingappa, Gustavo Delhon, Shi-Hua Xiang, Asit K. Pattnaik, David Steffen, Jay Reddy

**Affiliations:** 1 School of Veterinary Medicine and Biomedical Sciences, University of Nebraska-Lincoln, Lincoln, NE 68583 USA; 2 Nebraska Center for Virology, University of Nebraska-Lincoln, Lincoln, NE 68583 USA; 3 Present Address: Laboratory of Early Sickle Mortality Prevention, Cellular and Molecular Therapeutics Branch, National Heart, Lung, and Blood Institute, National Institutes of Health, Bethesda, MD 20892 USA; 4 Present Address: Division of Immuno-Oncology, CRISPR Therapeutics, Cambridge, MA 02139 USA; 5 Present Address: Aclaris Therapeutics, Inc., St. Louis, MO 63110 USA

**Keywords:** Live attenuated vaccines, Infection

## Abstract

Coxsackievirus B3 (CVB3), is commonly implicated in myocarditis, which can lead to dilated cardiomyopathy, in addition to causing acute pancreatitis and meningitis. Yet, no vaccines are currently available to prevent this infection. Here, we describe the derivation of a live attenuated vaccine virus, termed mutant (Mt) 10, encoding a single amino acid substitution H790A within the viral protein 1, that prevents CVB3 infection in mice and protects from both myocarditis and pancreatitis in challenge studies. We noted that animals vaccinated with Mt 10 developed virus-neutralizing antibodies, predominantly containing IgG2a and IgG2b, and to a lesser extent IgG3 and IgG1. Furthermore, by using major histocompatibility complex class II dextramers and tetramers, we demonstrated that Mt 10 induces antigen-specific T cell responses that preferentially produce interferon-γ. Finally, neither vaccine recipients nor those challenged with the wild-type virus revealed evidence of autoimmunity or cardiac injury as determined by T cell response to cardiac myosin and measurement of circulating cardiac troponin I levels, respectively. Together, our data suggest that Mt 10 is a vaccine candidate that prevents CVB3 infection through the induction of neutralizing antibodies and antigen-specific T cell responses, the two critical components needed for complete protection against virus infections in vaccine studies.

## Introduction

Enteroviral infections are common in humans worldwide ^1^. These are non-enveloped viruses consisting approximately 7.5 kb positive-sense, single-stranded RNA genome. They possess an icosahedral capsid consisting of 60 subunits with four structural viral proteins (VPs): VP1 to VP4. The coding region encodes a single polyprotein flanked by non-translated regions at both the 5′ and 3′ ends. Group A and group B coxsackieviruses belong to the genus *Enterovirus*, and several serotypes have been identified: 23 in group A and six in group B. Although some syndromes are caused only by group A coxsackieviruses, diseases like myocarditis and pancreatitis are caused mainly by group B coxsackieviruses^1,2^.

Within group B coxsackieviruses, coxsackievirus B (CVB)3 is commonly implicated in the causation of myocarditis^3^, in addition to causing acute pancreatitis^4^ and meningitis in those affected^5^. CVB-reactive antibodies have been detected in ~ 50% of dilated cardiomyopathy (DCM) patients, while enterovirus genomic material has been detected in up to 70%^3,6–9^, suggesting that CVB can be an important trigger of myocarditis/DCM and possibly involve autoimmune responses to cardiac antigens. Indeed, by using major histocompatibility complex (MHC) class II dextramers, we had previously demonstrated that A/J mice infected with CVB3 generate pathogenic myosin-specific T cells, suggesting that autoimmunity is an important sequalae of viral myocarditis^10^. While this observation raises concerns about the use of wild type (wt) viruses as vaccine candidates, successful derivation of altered viruses as vaccine candidates, free of virulence attributes and side effects like cardiac autoimmunity, is a challenging task in vaccine research.

In our efforts to analyze critical viral regions required for CVB3 to enter the target cells through coxsackievirus-adenovirus receptor (CAR) interaction, we noted one region corresponding to VP1 771–790 that participates in CAR binding. Recently, we created an infectious clone of CVB3 (pBRCVB3) whose ability to induce myocarditis was significantly attenuated, while occurrence of pancreatitis remained unaltered^11^. Using this virus as a backbone, we created a series of mutant viruses and identified one virus, termed Mutant (Mt) 10, that offered complete protection from both myocarditis and pancreatitis in challenge studies, suggesting that the Mt 10 virus could be used as a vaccine candidate. Mechanistically, the vaccine responses were accompanied by induction of both virus-neutralizing antibodies (nABs) and antigen-specific-T cell responses, with no evidence of autoimmunity or cardiac injury.

## Results

By generating a full-length infectious clone of the CVB3 genome (pBRCVB3), we had previously reported that the pBRCVB3 virus induces mainly pancreatitis, but its myocarditis-inducing ability was severely impaired^11^. Using this virus as the backbone, we sought to create attenuated mutant viruses that would lose their ability to induce both myocarditis and pancreatitis, thus leading us to identify one mutant virus possessing a mutation in the CAR-binding region as a vaccine candidate in the prevention of CVB3 infection.

### Localization of an epitope in the VP1 region that interacts with CAR

**Figure 1:**
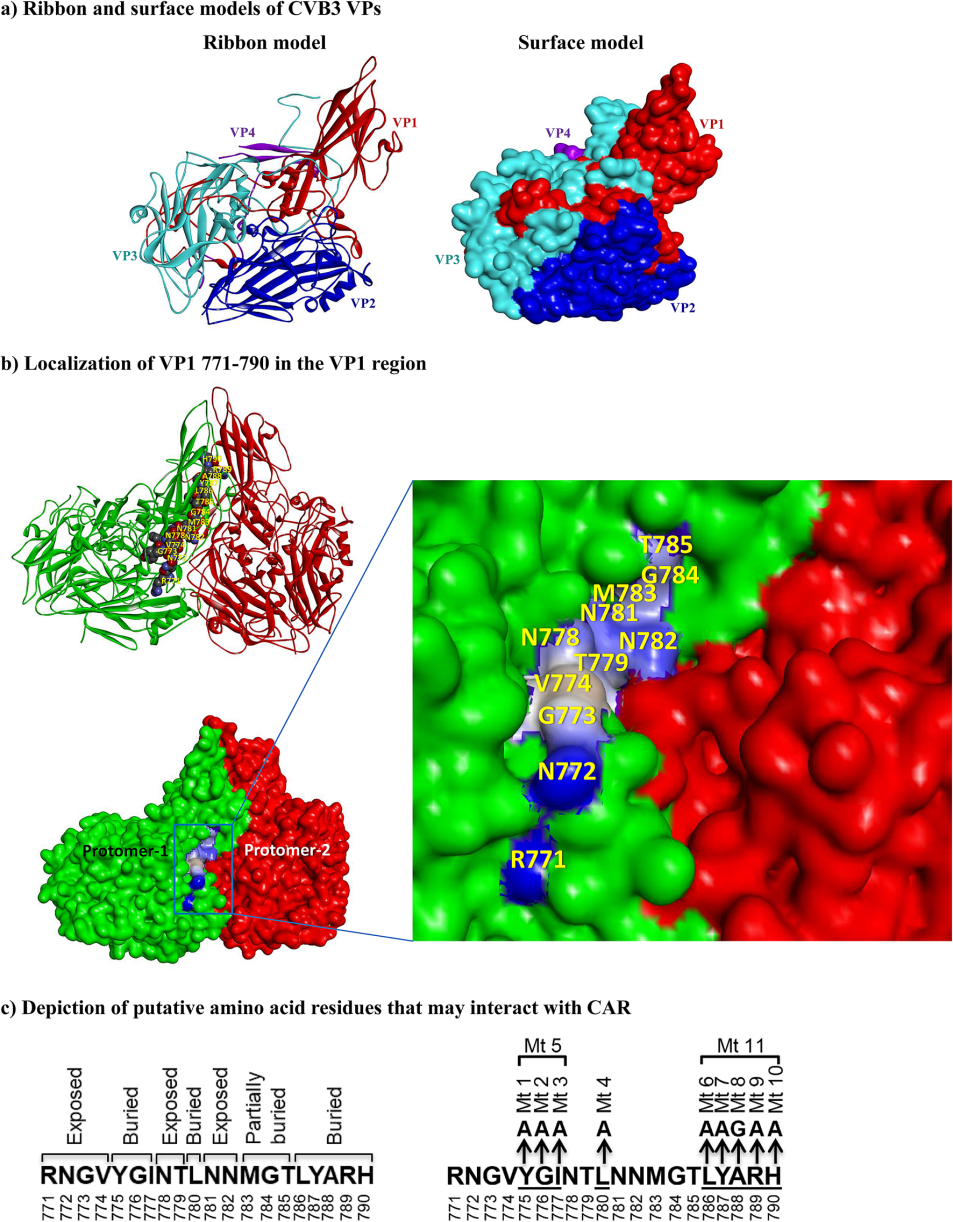
Structural characteristics of VP1 771–790 of CVB3 showing their relationship to the binding region between viral canyon and CAR, and an approach to create mutant viruses. (**a**) Ribbon and surface models of CVB3 VPs. Using the published pentamer structure of CVB3 (PDB: 1COV)^16,17^, all four structural proteins of CVB3 (VP1 [red], VP2 [blue], VP3 [turquoise], and VP4 [purple]) were localized in the protomer, as illustrated in the ribbon (left panel) and surface (right panel) models. (**b**) Localization of VP1 771–790 in the VP1 region. The amino acid residues of VP1 771–790, depicted in balls between two protomers (green and red) are shown in the top panel, and their positions are indicated in yellow. The surface model (bottom panel) shows the exposed amino acid residues that may potentially interact with the CAR; their positions are shown in yellow in the inset. The interactions of VP1 771–790 with CAR were analyzed using the BIOVIA Discovery Studio v4.0 (https://www.discngine.com/discovery-studio). (**c**) Depiction of putative amino acid residues that may interact with CAR. After localizing the residues within the VP1 region in relation to CAR as described above, the putative residues that potentially interact with the host receptor CAR were identified as exposed residues (RNGV, NT, and NN), whereas YGI, L, and LYARH were identified as buried residues, which are not expected to interact with CAR (left panel). The right panel represents mutations introduced at various positions individually or in combination.

Our initial efforts were intended to identify the immunogenic epitopes of CVB3 to track the generation of virus-specific T cell responses in infected animals^12^. We targeted VP1, one of the four structural proteins of CVB3 that has been identified as an immunogenic protein in other enteroviruses^13–15^. Using an overlapping peptide library, we identified three epitopes—VP1 681–700, VP1 721–740, and VP1 771–790—that induce antigen-specific, CD4 T cell responses as evaluated by MHC class II dextramers/tetramers^12^. Next, we took advantage of crystal structure data (PDB: 1COV)^16^ and, using the Discovery studio visualizer software, retrieved the footprints of the putative binding regions of CVB3 and CAR to determine the locations of viral epitopes. Using the cryo-electron microscopic model^17^, we mapped the VP1 to VP4 proteins as illustrated in the ribbon and surface models (Fig. 1a). These analyses led us to note that VP1 771–790 forms a part of the CAR-binding region within the canyon of CVB3 (Fig. 1b, top panel). Importantly, by analyzing the footprint of the CAR*-*CVB3-interacting area in the canyon, we noted that eight residues [arginine, asparagine, glycine, valine (RNGV); N, threonine (T), and NN; shown as balls] were exposed within VP1 771–790 (Fig. 1b, bottom panel with inset), whereas tyrosine, glycine, isoleucine (YGI), and leucine (L) of the N-terminal half and the C-terminal residues [leucine (L) to histidine (H)] were buried (not shown) in the peptide. Based on this information, we hypothesized that the exposed residues in the VP1 771–790 epitope are required for viral entry into the target cells through CAR interaction, whereas the buried residues are not critical for viral entry because they are not expected to interact with CAR (Fig. 1c). However, three residues toward the C-terminus, methionine (M; 783), G (784) and T (785), although buried partially, can also interact with CAR^17^ (Fig. 1b, bottom panel inset). Thus, we identified a region in CVB3 from which we would be able to manipulate the viral genome and derive mutant strains to test the hypothesis that the mutant viruses may retain infectivity, but lose pathogenicity and determine their phenotypes.

### Creation of CVB3 mutant viruses

To create the mutant strains, we used the pBRCVB3 virus^11^. We sought to generate mutant viruses by targeting nine buried residues within the VP1 771–790 region. These included VP1 775, 776 and 777, and 780, 786, 787, 788, 789 and 790, corresponding respectively to two stretches of amino acids, YGI and LYARH, including VP1 780 for L at the N-terminal end of the sequence (Fig. 1c and Table S1). Specifically, via PCR-based site-directed mutagenesis using primers (Table S2), each of the above amino acids were mutated to A, except for VP1 788 residue, which was mutated to G (Table S3). In addition, two more mutants were created, each representing mutations made in stretches from positions VP1 775 to VP1 777 and from VP1 786 to VP1 790 (Table S3). After generating plasmid constructs containing the mutated CVB3 genomes, locations of mutations were confirmed by Sanger DNA sequencing prior to virus recovery (Table S4).

### Recovery of infectious mutant viruses

**Figure 2:**
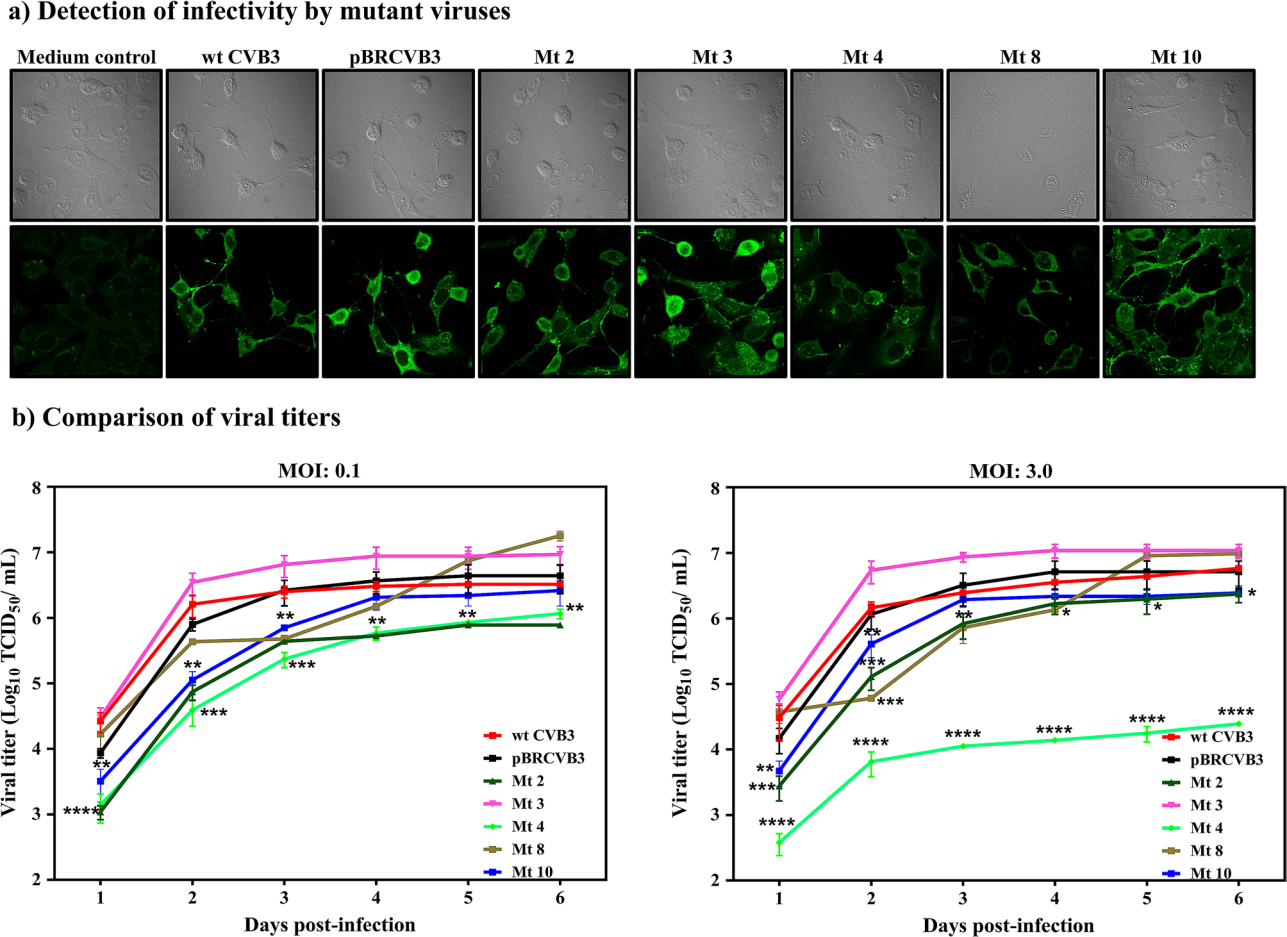
Recovery of infectious viruses from mutant clones. (**a**) Detection of infectivity by mutant viruses. Vero cells grown in monolayers on coverslips in 12-well plates were cultured in medium alone or infected with EMEM containing wt CVB3, pBRCVB3, Mt 2, Mt 3, Mt 4, Mt 8, or Mt 10, then incubated for 12 h at 37 °C. Cells were fixed and incubated with anti-CVB3 serum, and a secondary FITC-conjugated IgG was added to detect viral antigens. The coverslips were washed and mounted, then examined by Laser Scanning Confocal Microscope. Top panel: phase-contrast images. Bottom panel: immunofluorescence images. Original magnification: 60×. (**b**) Comparison of viral titers. Monolayers of Vero cells were grown to 80–90% confluence in 12-well plates. Triplicate wells were infected at an MOI of 0.1 or 3.0 with passage 2 viruses of wt CVB3, pBRCVB3, Mt 2, Mt 3, Mt 4, Mt 8, or Mt 10. After noting the CPE, the respective viral supernatants were harvested, and the viral titers (TCID_50_) were determined by Spearman-Karber method. Mean ± SEM values are shown (n = 3). Mann–Whitney test and Kruskal–Wallis test were used to determine significance between wt CVB3/pBRCVB3 and other mutant viruses at different times post-infection. *p ≤ 0.05, **p ≤ 0.01, ***p ≤ 0.001, and ****p ≤ 0.0001.

To recover infectious viruses, we performed transfection experiments in Vero cells using the in vitro-transcribed RNA obtained from each mutant clone. After passaging twice, we ascertained expression of viral proteins by immunofluorescence, using the wt CVB3 and pBRCVB3 as positive controls, with media containing uninfected cells serving as negative controls. As shown in Fig. 2a, cells infected with wt CVB3 or pBRCVB3 exhibited viral protein expression, but as expected, no expression was detected in mock-infected cells (medium control). By comparing the intensity of immunofluorescence, we noted that the cells transfected with viral RNAs representing the mutants 2, 3, and 10 had comparable expressions of viral proteins, whereas the fluorescence intensities for mutants 4 and 8 were relatively diminished (Fig. 2a). To rule out differences in transfection efficiency, we next determined the multi- and single-step growth kinetics of the mutant viruses using multiplicity of infection (MOIs) of 0.1, and 3.0 to ascertain if the mutations introduced in VP1 affect the growth of the mutant viruses. The analyses revealed significant differences between viruses, except for mutant 3. During the initial 3 days of infection at both MOIs, viral titers for mutants 2, 4, 8, and 10 were lower than wt CVB3 or pBRCVB3 viruses (0.8 to 2.2 log) (Fig. 2b). As the days progressed (days 4 to 6), viral titers continued to be low for mutants 2 and 4 (Fig. 2b, left panel), whereas those of mutants 8 and 10 reached the levels of wt CVB3 and pBRCVB3 viruses at MOIs 0.1 and 3.0. However, at MOI 3.0, no such difference was noted for mutant 2 (Fig. 2b, right panel). Of note, viral RNA obtained from other mutants—namely, Mt 1, Mt 5, Mt 6, Mt 7, Mt 9, and Mt 11—did not yield recoverable viruses, suggesting the mutations are lethal (data not shown). Together, the data suggested that the mutations introduced in positions 776 (Mt 2), 777 (Mt 3), 788 (Mt 8), and 790 (Mt 10), with an exception of 780 (Mt 4), did not appear to significantly affect viral replication, leading us to test their infectivity in vivo.

### Identification of Mt 10 as a vaccine candidate

**Figure 3:**
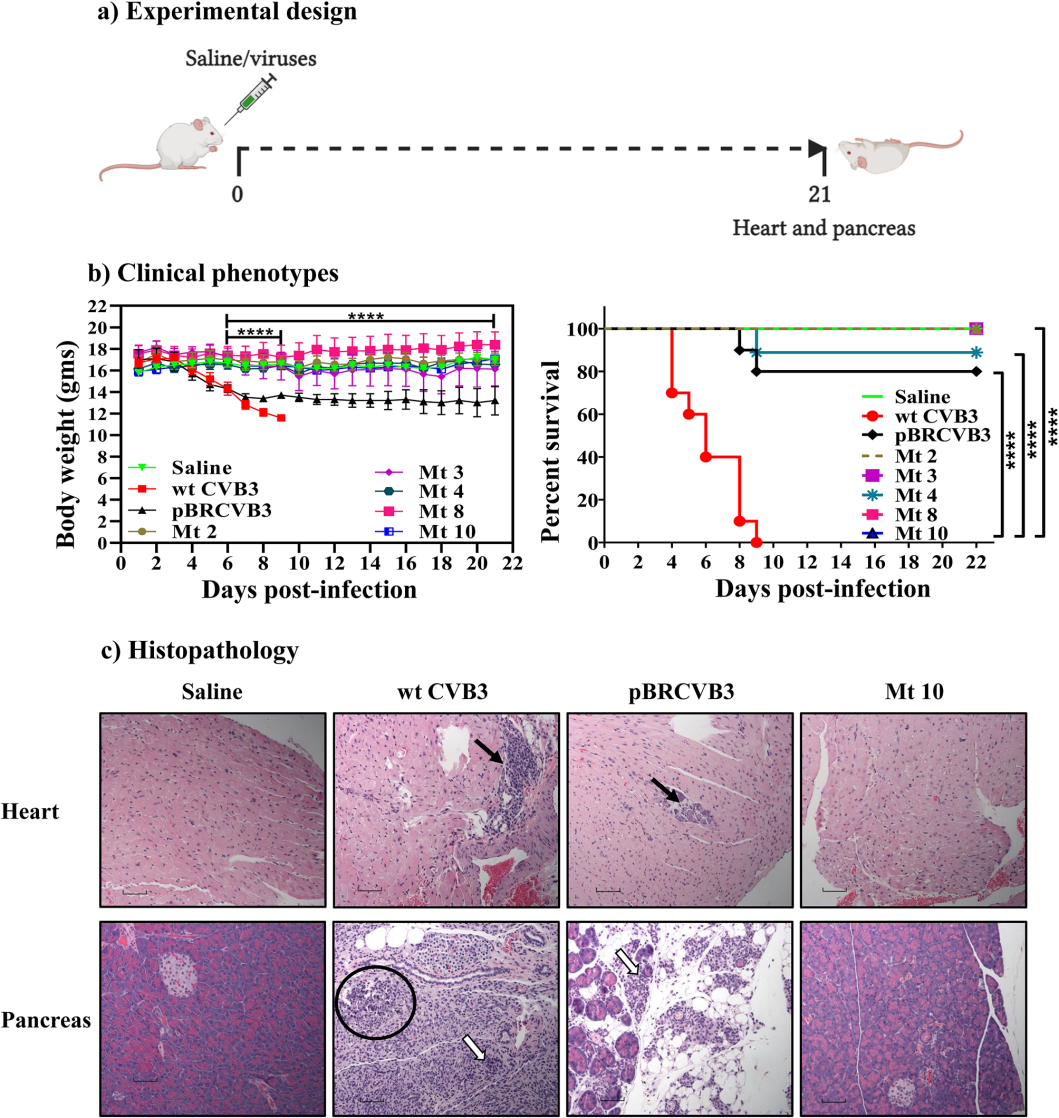
Disease phenotypes induced by various mutant viruses. (**a**) Experimental design. A/J mice were infected with wt CVB3, pBRCVB3, Mt 2, Mt 3, Mt 4, Mt 8, and Mt 10 viruses, with saline recipients as controls. At termination on day 21, heart and pancreas were collected for histology. (**b**) Clinical phenotypes. Body weights were taken every one to two days until termination and compared between groups (left panel); mortalities, if any, were noted to calculate survival rates (right panel). (**c**) Histopathology. Hearts and pancreata collected at termination on day 21 were processed for standard histology to evaluate inflammatory changes. Representative sections of hearts (top panel) and pancreata (bottom panel) from saline (negative control), wt CVB3 and pBRCVB3 (positive controls), and Mt 10 virus groups are shown. Solid and empty arrows represent infiltrations of MNCs in the heart and pancreatic sections, respectively. Circles indicate necrotic areas in the pancreatic sections. Magnification, 20×; scale bars, 20 µm. Data sets obtained from two individual experiments, each involving n = 4 to 5 mice, are shown. Two-way ANOVA with a Sidak’s post-test was used to compare body weight changes in saline group relative to wt CVB3/pBRCVB3 groups. Log-rank test with Bonferroni correction was used to compare survival curves. ****p ≤ 0.0001.

We infected groups of mice with five mutant viruses (Mt 2, Mt 3, Mt 4, Mt 8, and Mt 10) individually; at termination on day 21 post-infection, hearts and pancreata were collected for histology (Fig. 3a). In these experiments, wt CVB3 and pBRCVB3, and saline recipients were used as positive and negative controls, respectively. Figure 3b, left panel, shows that wt CVB3-infected mice, but not saline-recipients, had lost body weight by day 9 post-infection [p.i] (*p* ≤ 0.0001), whereas those infected with pBRCVB3 had a similar decline in relation to saline recipients (*p* ≤ 0.0001), but it occurred progressively as expected^11^. In contrast, animals infected with the mutant viruses remained clinically healthy and did not lose body weight compared to wt CVB3 or pBRCVB3 groups (*p* ≤ 0.0001), except for mice infected with Mt 3. We then compared mortalities between groups, and, expectedly, all animals infected with the wt CVB3 died by day 9 p.i (100%), as opposed to 20% (2/10) of those infected with pBRCVB3 virus (Fig. 3b, right panel, and Table 1, top panel). However, none of the animals infected with the mutant viruses died, except for one animal in the Mt 4 group (~ 11%) that had succumbed to the disease (Fig. 3b, right panel, and Table 1, top panel), suggesting that the degree of pathogenicity of mutant viruses may vary.

**Table 1:** Histological evaluation of hearts and pancreata in saline, wt CVB3, pBRCVB3, Mt 2, Mt 3, Mt 4, Mt 8, and Mt 10-infected mice.

Parameters	Saline	wt CVB3	pBRCVB3	Mt 2	Mt 3	Mt 4	Mt 8	Mt 10
**Heart**								
Incidence	0/9 (0.0)	10/10 (100.0)	8/10 (80.0)	0/9 (0.0)	2/9 (22.0)	3/9 (33.3)	1/16 (6.3)	0/9 (0.0)
Mortality	0/9 (0.0)	10/10 (100.0)	2/10 (20.0)	0/9 (0.0)	0/9 (0.0)	1/9 (11.1)	0/16 (0.0)	0/9 (0.0)
Myocardial lesions	0.0 (0.0)^a^	34.2 ± 13.4	11.8 ± 7.3	0.0 (0.0)^a^	0.2 ± 0.1^a^	0.4 ± 0.3^a^	0.1 ± 0.1^a^	0.0 (0.0)^a^
**Pancreas**								
Incidence	0/9 (0.0)^a^	10/10 (100.0)	10/10 (100.0)	8/9 (88.8)	9/9 (100.0)	5/9 (55.5)	5/16 (31.2)^b^	0/9 (0.0)^a^
Atrophy	0 (0.0)^a^	10/10 (100.0)	9/10 (90.0)	8/9 (88.8)	9/9 (100.0)	5/9 (55.5)	1/16 (6.3)^a^	0 (0.0)^a^
Inflammation	0 (0.0)^a^	10/10 (100.0)	10/10 (100.0)	8/9 (88.8)	9/9 (100.0)	5/9 (55.5)	5/16 (31.2)^b^	0 (0.0)^a^
Necrosis	0 (0.0)^a^	9/10 (90.0)	8/10 (80.0)	4/9 (44.4)	6/9 (66.6)	3/9 (33.3)^b^	0/16 (0.0)^a^	0 (0.0)^a^
Mineralization	0 (0.0)^a^	8/10 (80.0)	7/10 (70.0)	3/9 (33.3)^b^	6/9 (66.6)	2/9 (22.2)^b^	0/16 (0.0)^a^	0 (0.0)^a^

() Indicates percentages.

^a^Denotes significant differences in comparison with wt CVB3 and pBRCVB3 group (*p* < 0.001).

^b^Denotes significant differences in comparison with wt CVB3 and pBRCVB3 group (*p* < 0.05).

We then examined hearts and pancreata from the above groups for microscopic inflammatory changes. Expectedly, histological analyses revealed that 100% (10/10) of animals infected with the wt CVB3, and 80% (8/10) of animals infected with pBRCVB3 had myocardial lesions as indicated by inflammatory foci of 34.2 ± 13.4 and 11.8 ± 7.3, respectively (Table 1, top panel), with infiltrates primarily consisting of mononuclear cells (MNCs) (Fig. 3c, top panel). By comparison, isolated lesions were noted in heart sections only in groups that received Mt 3, Mt 4, and Mt 8, whereas sections from other groups (Mt 2 and Mt 10), including the saline group, were free of myocarditic lesions (Table 1, top panel). By analyzing pancreatic sections, we noted that mice infected with wt CVB3 or pBRCVB3 virus had comparable pancreatitis as revealed by atrophy, inflammation, necrosis, and mineralization (Fig. 3c, and Table 1, bottom panels); as expected, pancreatic sections from the saline group were devoid of such lesions. However, sections from groups infected with mutant viruses revealed incidence of pancreatitis to be similar for Mt 2 (~ 89%) or Mt 3 (100%) viruses, whereas incidence was relatively low in the Mt 4 virus group (~ 56%), followed by the Mt 8 virus group (~ 31%), but necrosis in the latter was also lacking (Table 1, bottom panel). Strikingly, however, sections from recipients of Mt 10 virus did not reveal pancreatitis, which is similar to sections from their healthy counterparts (Fig. 3c, and Table 1, bottom panels). Since animals infected with Mt 10 virus were free of both myocarditis and pancreatitis, we decided to evaluate whether the Mt 10 virus can be used as a vaccine candidate.

### Mt 10 virus offers protection against wt CVB3 virus in challenge studies

**Figure 4:**
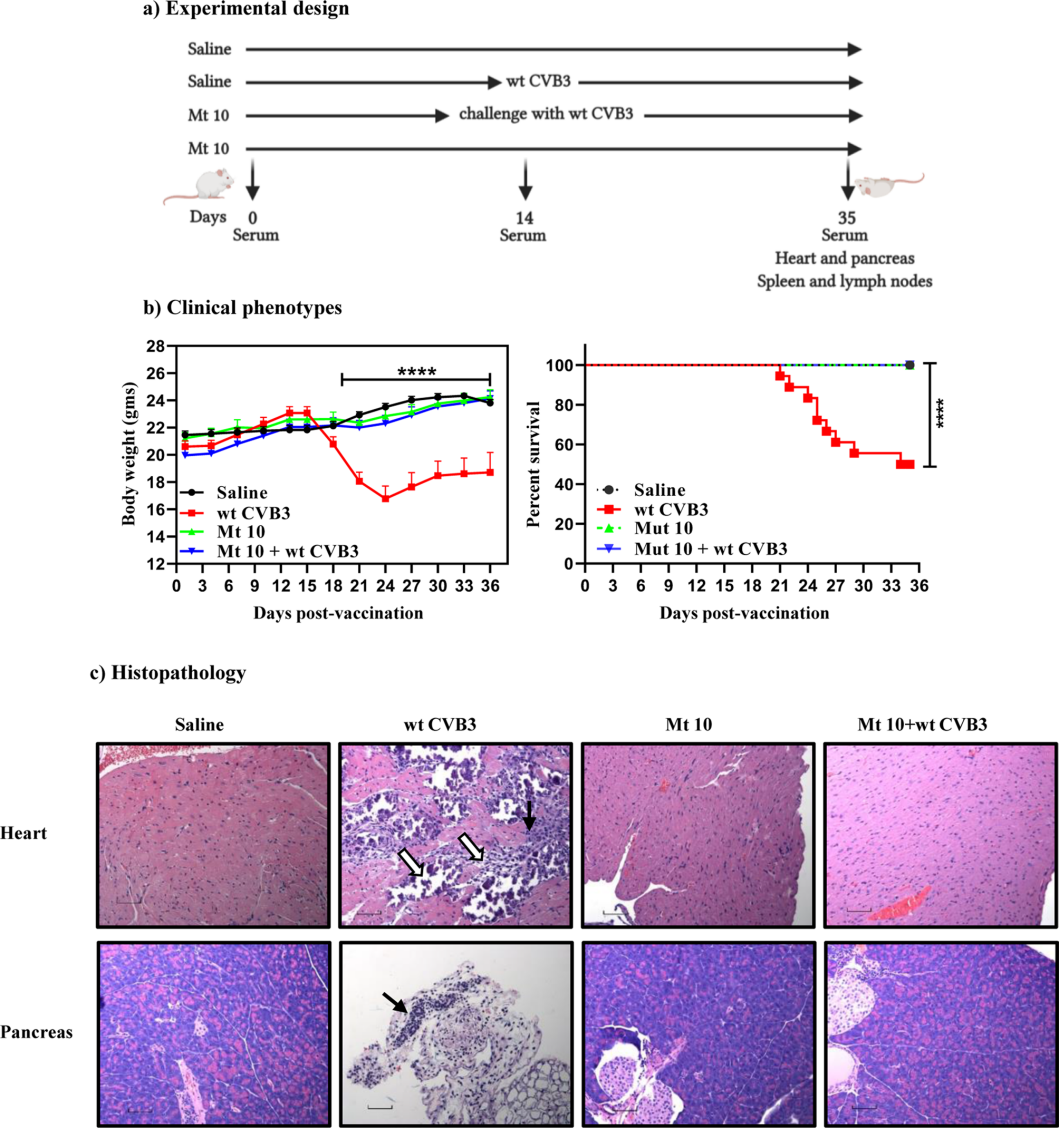
Mt 10 virus offers protection against wt CVB3 infection in challenge studies. (**a**) Experimental design. Two groups of mice each were administered saline or Mt 10 virus on day 0 after serum was collected. After 14 days, serum was collected, and one group from each was challenged with CVB3. Experiments were terminated 21 days later, and serum and tissues were collected for in vitro experimentation. (**b**) Clinical phenotypes. Body weights (left panel) and survival rates (right panel) between different groups are shown. (**c**) Histopathology. Hearts and pancreata collected from the indicated groups and processed by H and E staining to evaluate inflammatory changes. Representative heart (top panel) and pancreatic (bottom panel) sections are shown. Heart sections from wt CVB3 group shows infiltrations (solid arrow) and mineralization, necrosis, and fibrosis (empty arrow). Pancreatic sections from wt CVB3 group had infiltrations (solid arrow) as opposed to normal sections in the saline, Mt 10 virus, and Mt 10 virus/wt CVB3-challenged groups. Magnification, 20×; scale bars, 20 µm. Data sets obtained from three individual experiments, each involving n = 3 to 8 mice, are shown. Two-way ANOVA with a Sidak’s post-test was used to compare body weight changes in vaccine and saline groups relative to wt CVB3 group. Log-rank test with Bonferroni correction was used to compare survival curves. ****p ≤ 0.0001.

We performed challenge studies with an expectation that animals primed with Mt 10 virus would be protected from wt CVB3 challenge. We tested this hypothesis by immunizing animals with a single dose of Mt 10 virus and challenging them 14 days later with wt CVB3. After 21 days post-challenge, animals were euthanized and tissues were collected for histopathology. Saline groups were used as controls (Fig. 4a). Clinically, non-vaccinated animals infected with wt CVB3 virus lost body weight significantly starting ~ day 3 post-infection. Similar to the saline control, animals in the Mt 10 vaccine group, and more importantly, animals in the Mt 10 + wt CVB3 group did not lose body weight and remained clinically healthy during the length of the experiment (*p* ≤ 0.0001) (Fig. 4b, left panel). These observations were further captured by analyzing mortality rates; 50% (9/18) of animals in the wt CVB3 alone group died, but no mortalities were noted in saline, Mt 10-alone, and Mt 10/wt CVB3-challenged groups (Fig. 4b, right panel, and Table 2, top panel). Additionally, we did not observe sex-based differences with vaccine protection, but it was critical to evaluate tissues for inflammatory changes, if any, in the challenged animals.

**Table 2:** Histological evaluation of hearts and pancreata in saline, wt CVB3, Mt 10-infected, and Mt 10-infected and challenged mice.

Parameters	Saline	wt CVB3	Mt 10	Mt 10 + wt CVB3
**Heart**				
Incidence	0/9 (0.0)	12/18 (66.7)	0/16 (0.0)	0/17 (0.0)
Mortality	0/9 (0.0)	9/18 (50)	0/16 (0.0)	0/17 (0.0)
Myocardial lesions	0.0 (0.0)^a^	34.5 ± 13.3	0.0 (0.0)^a^	0.0 (0.0)^a^
**Pancreas**				
Incidence	0 (0.0)	16/18 (88.9)	1/16 (6.2)	0 (0.0)
Atrophy	0 (0.0)^a^	16/18 (88.9)	1/16 (6.2)^a^	0 (0.0)^a^
Inflammation	0 (0.0)^a^	16/18 (88.9)	0/16 (0.0)^a^	0 (0.0)^a^
Necrosis	0 (0.0)	0/18 (0.0)	0/16 (0.0)	0 (0.0)
Mineralization	0 (0.0)	0/18 (0.0)	0/16 (0.0)	0 (0.0)

() Indicates percentages.

^a^Denotes significant differences in comparison with wt CVB3 group (*p* < 0.0001).

Histologically, heart sections from ~ 67% (12/18) of mice infected with wt CVB3 had myocardial lesions (34.5 ± 13.3), whereas inflammatory changes were lacking in both vaccine-alone and vaccine/challenged groups (Fig. 4c and Table 2, top panel). Similarly, 89% (16/18) of animals infected with wt CVB3 had pancreatitis as indicated by atrophy (89%) and inflammation (89%), whereas sections from the vaccine or vaccine/challenged groups did not reveal such changes (Fig. 4c and Table 2, bottom panel), except that a small area of atrophy was noted in only one animal in the vaccine group (Table 2, bottom panel). Overall, the finding that the vaccine recipients challenged with wt CVB3 were completely protected from both myocarditis and pancreatitis implies that disease protection might have been mediated by the Mt 10 virus. Thus, we identified Mt 10 as an attenuated, avirulent CVB3 vaccine virus, but the data raised questions as to the underlying mechanisms of disease protection.

### Mt 10 virus induces virus nABs predominantly of IgG isotypes

**Figure 5:**
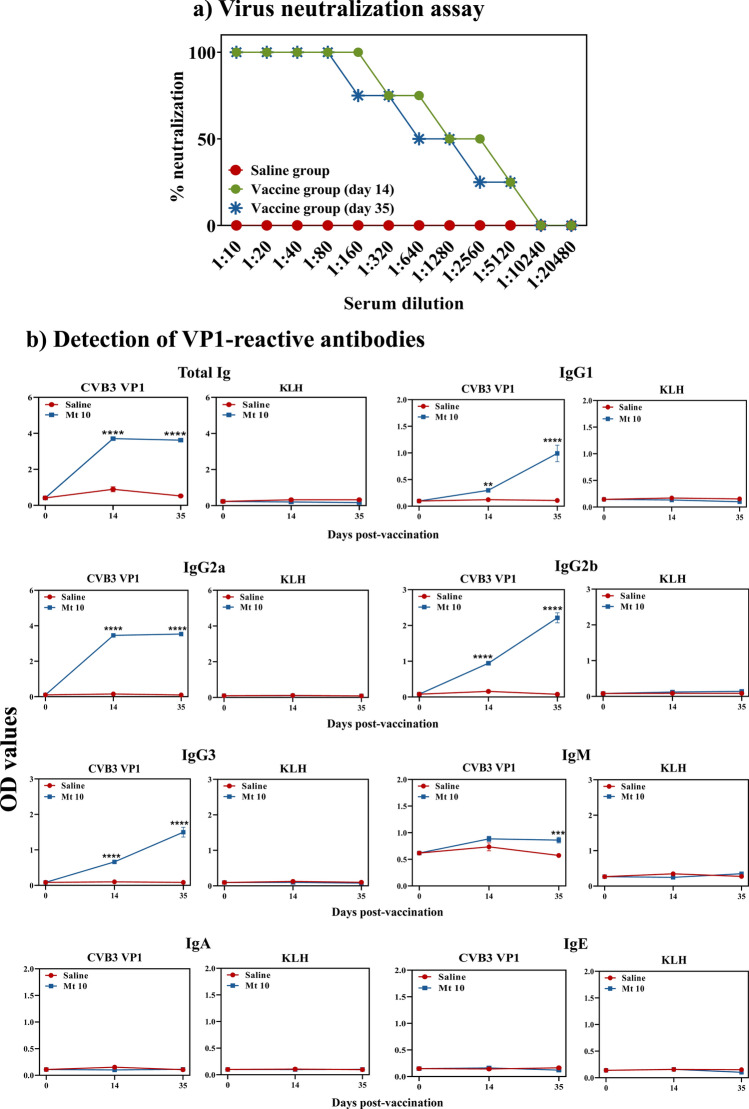
Mt 10 virus induces neutralizing antibodies of IgG isotypes. (**a**) Virus neutralization assay. Sera were obtained from saline and Mt 10 virus groups on days 14 and 35, and after diluting serially, samples were incubated with wt CVB3. The mixtures were transferred to plates containing Vero cells, and incubation was continued for four days to calculate the percentage neutralization based on CPE. Data from n = 6 samples, each representing a pool of sera from 3 to 5 mice, are shown. (**b**) Detection of VP1-reactive antibodies. Serum samples collected from the above groups were diluted (1:150) and added in duplicates to high-binding plates previously coated with CVB3-VP1 or KLH (control). After adding HRP-conjugated goat anti-mouse total Ig, IgG1, IgG2a, IgG2b, IgG3, IgM, IgA, and IgE as detection antibodies, reactions were stopped. Plates were read at 450 nm to obtain the OD values. Mean ± SEM values obtained from n = 6 samples, each representing 3 to 5 mice, are shown. Mann–Whitney test was used to determine significance between groups. **p ≤ 0.01, ***p ≤ 0.001, and ****p ≤ 0.0001.

An important component of the adaptive immune response is the production of antibodies that are critical to preventing colonization and spread of infections. First, we measured nABs based on cytopathic effect (CPE), which involved assessment of percent neutralization of wt CVB3 in Vero cells exposed to serum collected from saline or Mt 10 vaccinated groups. Figure 5a shows that the virus was not neutralized when cells were exposed to serum from saline recipient animals, as expected. In contrast, sera harvested from vaccine recipients on day 14 showed 100% virus neutralization at 1:160 dilution, and virus neutralization of ~ 50% or more was still evident at dilutions up to 1:2560. Similar trends were observed with sera collected at day 35 post-vaccination, but no striking differences were noted between days 14 and 35 post-vaccination (Fig. 5a). While these data indicated that Mt 10 virus has the ability to induce nABs, the possibility remained that qualitative variations may exist between different antibody isotypes specific to the virus.

To that end, we measured total Ig, IgG1, IgG2a, IgG2b, IgG3, IgM, IgA, and IgE by enzyme-linked immunosorbent assay (ELISA) using CVB3 VP1 as a specific viral antigen, with Keyhole limpet hemocyanin (KLH) serving as a negative control, and we made a few observations (Fig. 5b). (i) Serum from both saline and Mt 10 groups did not react to KLH for any of the isotypes indicated above, ruling out the possibility of non-specific reactivity. (ii) Reactivity of sera for IgE and IgA, and to a lesser extent for IgM except at day 35, was lacking for VP1 in the vaccine group, suggesting that these isotypes are not the major components of the antibody response against the virus. (iii) Vaccine recipients had elevated levels of VP1-reactive total Igs by day 14 post-vaccination, and their levels remained elevated up to day 35. (iv) As to the various IgG isotypes, they were elevated only in the vaccinated groups, but not in controls, and occurred in the order of IgG2a >  > IgG2b > IgG3 > IgG1 (Fig. 5b). Since vaccine-induced antibody responses were dominated by IgG isotypes, the data pointed to a role for simultaneous induction of virus-specific T cell responses as T cell-derived cytokines are critical for isotype switching^18^.

### T cell responses induced by Mt 10 virus are antigen-specific and involve mainly Th1 cytokines

**Figure 6:**
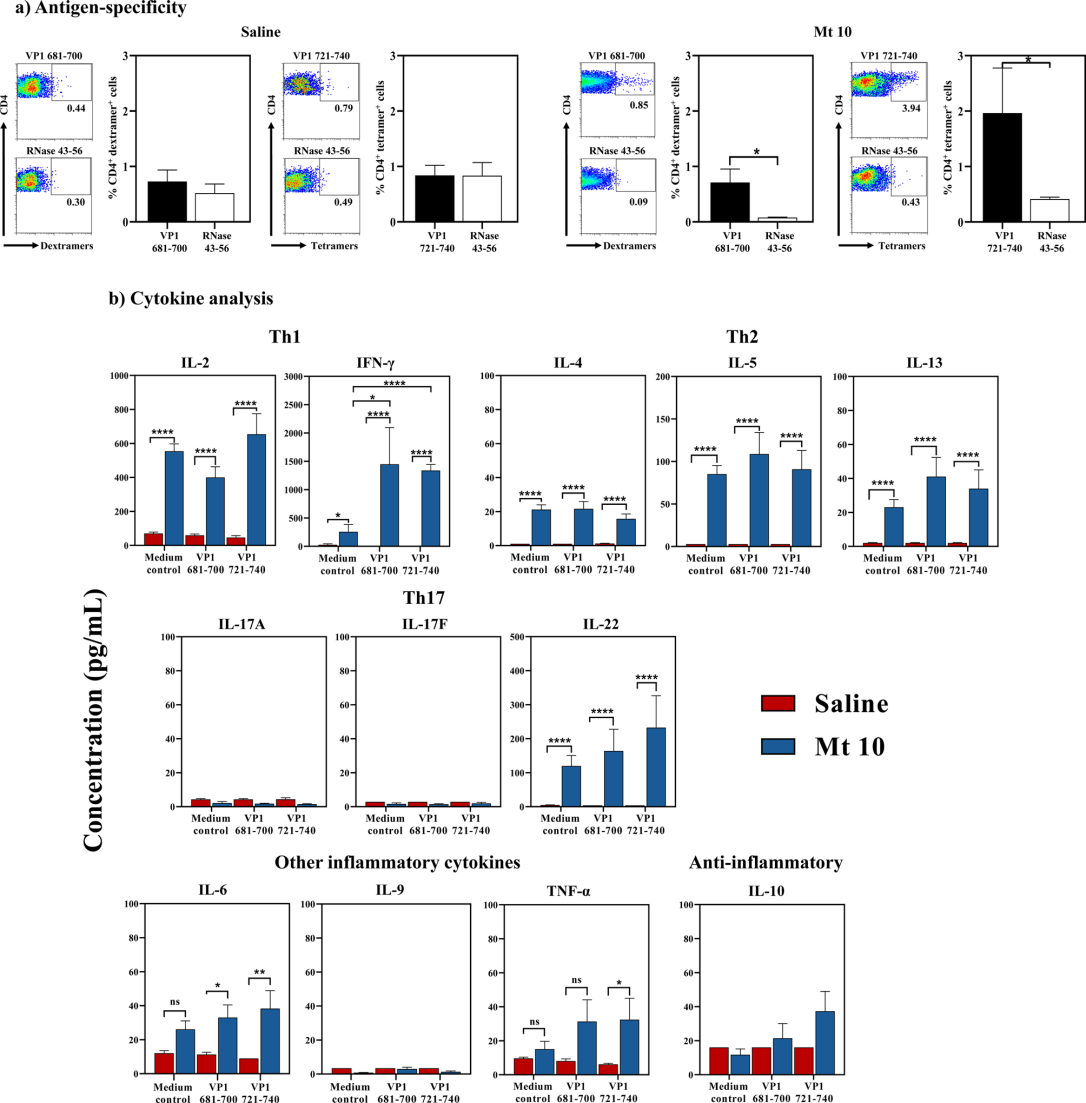
Mt 10 virus induces the generation of antigen-specific, T cells capable of producing predominantly, IFN-γ. (**a**) Antigen-specificity. Groups of mice were injected with saline or Mt 10 virus, and 35 days later, lymphocytes were prepared. Cells were stimulated with VP1 681–700 or 721–740 and maintained in IL-2 medium. Cells harvested between days, 7–10 post-stimulation were stained with the indicated IA^k^/tetramers and dextramers, anti-CD4 and 7-AAD. After acquiring the cells by flow cytometry, tetramer and dextramer ^+^ cells were analyzed in the live (7-AAD¯) CD4 subset using FlowJo software. RNase 43–56, control. Representative flow cytometric dot-plots and the mean ± SEM values from three individual experiments, each involving n = 3 to 8 mice are indicated. (**b**) Cytokine analysis. Supernatants harvested from the above cultures on day 3 post-stimulation were analyzed for cytokines by LEGENDplex cytokine bead array as described in the methods section. Mean ± SEM values obtained from three individual experiments, each involving n = 3 to 8 mice are indicated. Unpaired Student’s t-test (two-tailed) was used to determine significance between groups [for panels (**a**,**b**)]. *p ≤ 0.05, **p ≤ 0.01, and ****p ≤ 0.0001.

We recently reported identification of two major CD4 T cell epitopes within CVB3 VP1, VP1 681–700, and VP1 721–740, and construction of MHC class II tetramers and dextramers to evaluate their antigen-specificity^12^. Using these tools, we analyzed virus-specific T cells in animals injected with saline or vaccinated with Mt 10 virus. In brief, a pool of spleens and lymph nodes, hereafter termed lymphocytes, harvested from these groups were stimulated with VP1 681–700 or VP1 721–740 peptides, and staining was performed by flow cytometry using IA^k^/VP1 681–700 dextramers and VP1 721–740 tetramers and their corresponding controls (Bovine ribonuclease [RNase] 43–56)^12^. As expected, CD4 T cells from saline-recipient animals did not bind VP1 681–700 or VP1 721–740 dextramers or tetramers, and their staining intensities were comparable to those of RNase 43–56 (control) (Fig. 6a, left panel). Similar analysis in vaccine recipients revealed staining of CD4 T cells for both VP1 681–700 dextramers (0.7 ± 0.2; *p* ≤ 0.05) and VP1 721–740 tetramers (1.9 ± 0.8; *p* ≤ 0.05), whereas staining for RNase 43–56 was negligible (Fig. 6a, right panel). While these data lend support for the occurrence of antigen-specific T cell expansion in response to vaccine virus, additional studies were needed to investigate the nature of cytokine responses.

**Figure 7:**
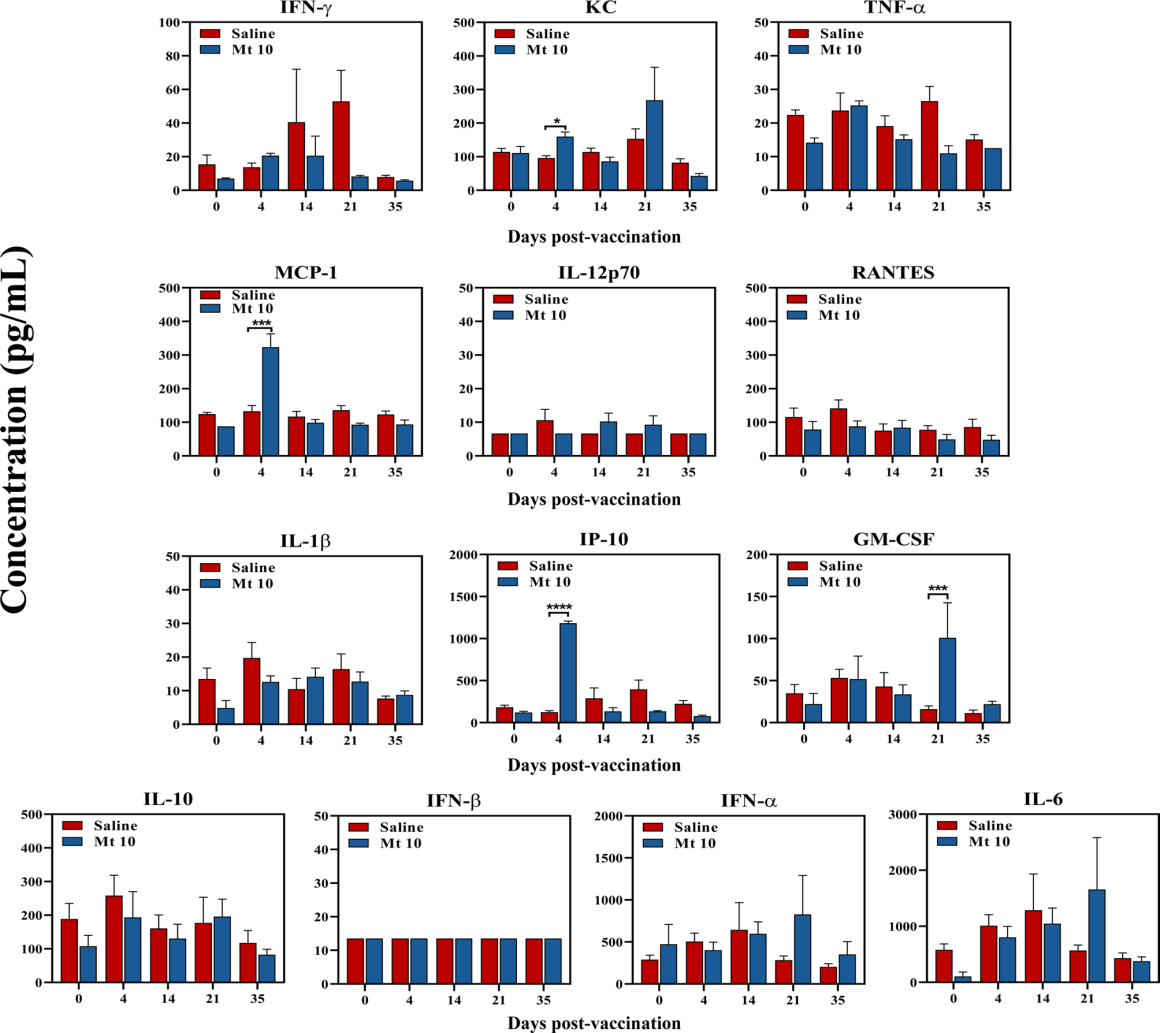
Analysis of anti-viral cytokines and chemokines in response to Mt 10 virus. Groups of mice were administered with saline or Mt 10 virus, and sera were collected periodically from day 0 to day 35. Samples were processed for a panel of indicated anti-viral cytokines and chemokines by LEGENDplex cytokine bead array as described in the methods section, and their levels were compared. Mean ± SEM values obtained from three individual experiments, each involving n = 3 to 8 mice are indicated. Unpaired Student’s t-test (two-tailed) was used to determine significance between groups. *p ≤ 0.05, ***p ≤ 0.001, and ****p ≤ 0.0001.

We adopted multiplex bead array analysis to analyze cytokine responses in culture supernatants harvested from antigenic stimulations described above for a panel of T helper (Th) 1, Th2, and Th17, including interleukin (IL)-6, tumor necrosis factor (TNF)-α, and anti-inflammatory (IL-10) cytokines. The analyses revealed detection of mainly interferon (IFN)-γ and IL-2 [Th1], followed by IL-22, representative of Th17 subset, and IL-5, IL-4, and IL-13 (Th2) in cultures derived from vaccine-recipients as compared to saline-recipient animals without antigenic stimulations, suggesting that the virus-primed lymphocytes can spontaneously produce these cytokines (Fig. 6b). While IL-9, IL-17A, and IL-17F were consistently absent, other inflammatory cytokines (IL-6 and TNF-α) were also detected in low amounts in vaccine-recipients with a tendency for IL-10 to be high in the vaccine groups (Fig. 6b). However, upon stimulation with viral peptides (VP1 681–700 and VP1 721–740), we noted upregulation of only IFN-γ, indicating that the VP1-specific T cells capable of producing IFN-γ can form one component of virus-reactive cells. It is possible that the virus-sensitized cells may contain T cell specificities for other viral antigens that we have not examined in this study. Furthermore, we also examined a panel of anti-viral cytokines in serum samples collected at various time points (from day 0 to day 35) post-vaccination. These analyses revealed significant elevation of chemokines such as IFN-γ -induced protein (IP)-10 (*p* ≤ 0.0001), monocyte chemoattractant protein (MCP)-1 (*p* ≤ 0.001), and keratinocyte-derived chemokine (KC) (*p* ≤ 0.05) on day 4 after vaccination, whereas granulocyte–macrophage colony-stimulating factor (GM-CSF) (*p* ≤ 0.001) was detected at day 21 when compared with saline recipients (Fig. 7). In contrast, other cytokines (IFN-γ, TNF-α, IL-12p70, Regulated upon Activation, Normal T Cell Expressed and Presumably Secreted [RANTES], IL-1β, IL-10, IFN-α, IFN-β, and IL-6), although detected in varied amounts, were not significant between groups (Fig. 7). Taken together, our data suggest that the Mt 10 virus can induce antigen-specific T cell responses capable of producing mainly IFN-γ, a critical cytokine in fighting intracellular pathogens like viruses, in addition to promoting other anti-viral cytokines described above. While these components together with virus nABs might have contributed to disease protection mediated by the Mt 10 virus, safety concerns, if any, needed to be evaluated.

### Animals vaccinated with Mt 10 virus did not reveal autoimmune response and cardiac injury

**Figure 8:**
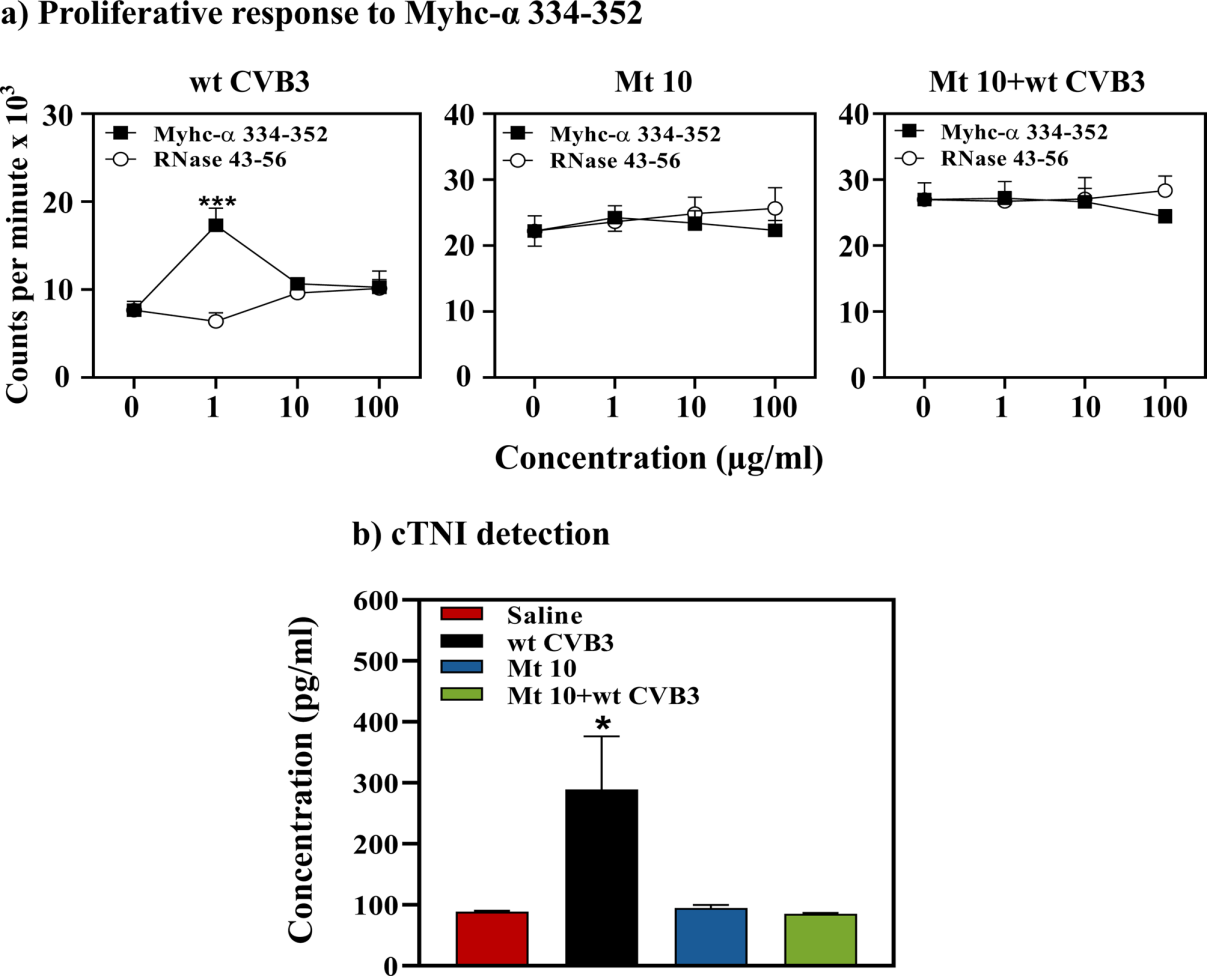
Mice injected with Mt 10 virus did not reveal autoimmunity or cardiac injury. (**a**) Proliferative response to Myhc-α 334–352. Groups of mice were administered saline or Mt 10 virus, and after 14 days, they were challenged with or without CVB3. Three weeks later, lymphocytes were prepared, and cells were stimulated with or without Myhc-α 334–352 or control (RNase 43–56) for 2 days. After pulsing with ^3^[H] thymidine for 16 h, proliferative responses were measured as cpm. Mean ± SEM values obtained from three individual experiments, each involving n = 3 to 8 mice, are shown. (**b**) cTnI detection. Undiluted sera obtained from the above groups were added in duplicates to pre-coated, cTnI ELISA strip plates. After incubation and a series of washes, HRP-conjugated rabbit anti-mouse antibody was added followed by TMB substrate and stop solution. Plates were read at 450 nm to obtain OD values, and concentrations of unknown samples were determined using the standard curve. Mean ± SEM values representing 6 samples per group, each containing n = 3 to 5 mice, are shown. Unpaired Student’s t-test (two-tailed) was used to determine significance between groups [for panels (**a**,**b**)]. *p ≤ 0.05, and ***p ≤ 0.001.

We had previously reported that CVB3 infection can lead to the induction of pathogenic T cells reacting to cardiac myosin as a secondary event^10,19^. To ensure that the Mt 10 virus is safe and does not induce side effects, we considered two readouts: assessment of cardiac myosin-reactive T cells to determine autoimmunity^20^, and measurement of cardiac troponin I (cTnI) as a cardiac injury marker^21^. Using T-cell proliferation assay, we noted myosin reactivity in animals infected with the wt CVB3, as indicated by the appearance of T cells responding to cardiac myosin heavy chain (Myhc)-α 334–352 (*p* ≤ 0.001) (Fig. 8a, left panel), an immunodominant epitope that induces autoimmune myocarditis in A/J mice^10,20^. Such reactivity was lacking in both vaccine-recipients (Fig. 8a, middle panel) and those challenged with wt CVB3 (Fig. 8a, right panel). Under similar conditions, we analyzed sera samples by ELISA for cTnI. As shown in Fig. 8b, cTnI was detected only in the wt CVB3-infected group (*p* ≤ 0.05), but not in the vaccine or vaccine/challenge groups. Taken together, the data suggest that the Mt 10 virus, despite being live attenuated, is unlikely to induce side effects described above, since cardiac injury is expected to occur as a consequence of direct virus-induced damage to the cardiac tissue^22–24^.

### Vaccine-recipients are free of wt CVB3 in challenge studies

**Figure 9:**
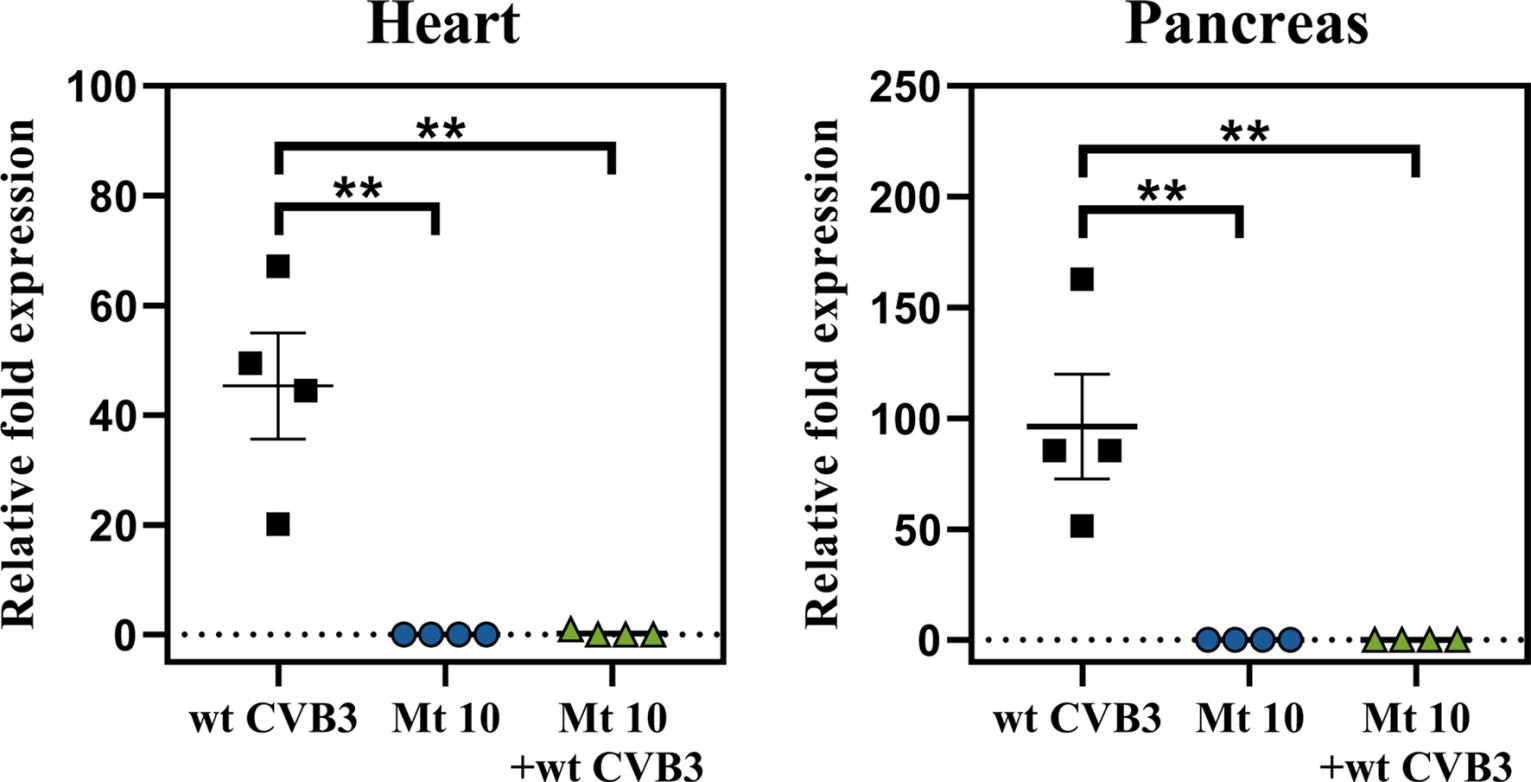
Recipients of Mt 10 virus challenged with the wt CVB3 virus did not reveal viral RNA. Groups of mice were administered saline or Mt 10 virus, and 14 days later, they were challenged with or without wt CVB3. Three weeks post-challenge, hearts and pancreata were collected, total RNA was extracted, and CVB3 RNA was estimated by quantitative PCR targeting VP1 sequences, as described in the methods section. After normalizing the expression levels viral RNA relative to GAPDH, 2^−(∆∆*C*t)^ values were calculated. Mean ± SEM values representing 4 samples per group, each containing n = 3 to 5 mice, are shown. Mann–Whitney test was used to determine significance between groups. **p ≤ 0.01.

The disease course induced by CVB3 in infected mice assumes viral and non-viral phases that occur in continuum, with the possibility that viral nucleic acids can be present in chronically infected animals^25^. We sought to evaluate this phenomenon by looking for viral RNA in hearts and pancreata harvested from animals inoculated with wt CVB3, vaccine alone, and vaccine-challenged groups. This was achieved by quantitative/real-time RT-PCR using the probes and primers specific to CVB3-VP1. Expectedly, hearts and pancreata obtained from the wt CVB3 alone group contained CVB3 viral nucleic acids (*p* ≤ 0.01) (Fig. 9). Importantly, viral RNA was not detected in the vaccine group, including animals challenged with the wt CVB3 (Fig. 9), suggesting that immune responses generated by the Mt 10 virus may be sufficient to prevent infection by the wt CVB3.

## Discussion

In this report, we describe the creation and characterization of an avirulent strain of CVB3 as a live attenuated vaccine to prevent both myocarditis and pancreatitis induced by the wt CVB3 virus. Experimentally, various vaccine strategies have been tested with varied efficacies^26–33^. But, none of these approaches evaluated for their ability to prevent both myocarditis and pancreatitis^31–33^ except one modified live mutant virus (CVB3/H3)^34^, but in the latter, viral titers were still present in the hearts. Additionally, side effects such as autoimmunity were also not investigated^34,35^ since attenuated vaccine strains may retain a low degree of virulence, causing tissue damage in target organs such as heart, leading to autoimmunity as we have previously reported with the wt CVB3^10,19^. Furthermore, to effectively prevent viral infections, induction of both antibody and T cell responses is critical because antibodies are needed to neutralize the viruses, and elimination of established infections requires participation of T cells. Therefore, ideally, vaccines are expected to induce both antigen-specific antibody and T cell responses and be free of side effects such as autoimmunity.

Our initial objective was to identify immunogenic T cell epitopes for analyzing virus-reactive T cell responses in a broad-spectrum of CVB infections. In that direction, we had successfully identified three T cell epitopes (VP1 681–700, VP1 721–740, and VP1 771–790) and demonstrated that two of these (VP1 681–700 and VP1 721–740) could be used to analyze antigen-specific T cell responses in multiple CVB serotypes^12^. However, by localizing these epitopes within the viral canyon, we observed that VP1 771–790 had the potential to interact with the CAR, one of the receptors needed for viral entry into target cells^36^. Of note, virus entry requires attachment of CVB3 to CD55 (also called decay accelerating factor) which then facilitates interaction with the CAR^37,38^. Furthermore, interaction of CAR with CVB3, but not with CD55 alone leads to lytic infection^39^. Since, we wanted to preserve the virus entry to be able to induce immune responses by unleashing the critical viral proteins, but not lytic infection leading to tissue injury, we chose not to target and disrupt the virus attachment to CD55. As illustrated (Fig. 1), VP1 771–790 contains 9 buried and 11 exposed amino acid residues. We hypothesized that by mutating the buried residues within VP1 771–790, virus infectivity could still be maintained, since the exposed residues required for CAR interaction would not be altered. As a result, virus could enter the target cells, and viral proteins could be expected to be presented to the immune system. Coincidentally, however, we had identified a variant of wt CVB3 (pBRCVB3) and demonstrated that pBRCVB3 could induce mainly pancreatitis, while its myocarditis-inducing ability was impaired^11^. Thus, we sought to create mutant viruses using the genomic sequence of pBRCVB3 as a backbone (Fig. 1), expecting one or more of the mutant strains to yield infectious viruses for infection studies to determine their pathogenic mechanisms, if any, related to the heart vs. pancreas.

Our approach to create mutant viruses was unique in that we specifically targeted the buried amino acids of the viral capsid canyon, which are less likely to interact with the CAR, leading to the recovery of infectious viruses from five clones, Mt 2, Mt 3, Mt 4, Mt 8, and Mt 10 (Fig. 2). Lack of virus recovery for clones possessing mutations at positions 775 (Mt 1), 786 (Mt 6), 787 (Mt 7), and 789 (Mt 9), which are also buried residues, suggested that these amino acids may take part in viral entry and/or are indispensable for viral replication. It is also possible that mutations of some of the buried residues may have altered the local structure at the canyon floor so that the viruses are unable to interact with the CAR receptor, rendering them noninfectious. Further work is needed to test these possibilities. Similarly, we failed to recover infectious viruses from clones bearing stretches of 3–5 substitutions in the buried residues, indicating that viruses appear not to tolerate multiple mutations. Nonetheless, successful recovery of mutant viruses from the five clones described above provided us an opportunity to investigate their disease-inducing potential in vivo.

From infection studies, it was clear that only one (Mt 10) of the five mutant viruses failed to induce both myocarditis and pancreatitis, whereas others (Mt 2, Mt 3, Mt 4 and Mt 8) induced mild myocarditis, if any; however, infected animals consistently developed pancreatitis, except that incidence and severity of pancreatitis induced with the Mt 8 virus were low (Table 1). Attenuated myocarditis induced by these mutant viruses was not surprising because all mutant viruses were created using the genomic sequence of pBRCVB3, and we had previously demonstrated that the infectious virus derived from this clone (pBRCVB3) could mainly induce pancreatitis, but not myocarditis^11^. Thus, lack of pancreatitis in animals infected with only Mt 10 virus could mean that the amino acid residue at position 790 may be critical to induce pancreatitis, either because Mt 10 cannot colonize the pancreas or, alternatively, lose pathogenicity in that organ. Similar attenuated viruses, including enteroviruses, have been investigated as vaccine candidates for a number of diseases^40–42^.

By investigating the vaccine characteristics of Mt 10 virus, we made three observations. First, priming animals with a single dose of the Mt 10 virus was sufficient to prevent infection by a lethal dose of wt CVB3 in challenge studies, and infected animals developed neither myocarditis nor pancreatitis (Fig. 4 and Table 2). Second, mechanistically, animals injected with Mt 10 virus develop nABs predominantly containing IgG antibodies of IgG2a and IgG2b isotypes, followed by those of IgG3 and IgG1 isotypes (Fig. 5). Likewise, Mt 10 recipients developed virus-specific T cell responses producing mainly IFN-γ (Fig. 6), in addition to anti-viral cytokines. Generation of both virus-reactive antibodies and T cells was not surprising because live attenuated viruses are expected to induce both responses^43^. Furthermore, by relating various antibody isotypes and cytokine responses, an association was evident between IgG2a and IFN-γ response, as the latter is needed for switching to IgG2a and IgG3^44,45^. However, reports indicate that IFN-γ can downregulate IgG2b^46^, but its detection in the recipients of Mt 10 virus may mean that other cytokines not examined in this study might have been involved in this process. One such cytokine is transforming growth factor-β, which is a known inducer of IgG2b^47^. Similarly, IL-4, IL-5, IL-13 (Th2), and IL-22 (Th17) were elevated in culture supernatants from vaccine recipients (Fig. 6). Reports indicate that IL-4 promotes IgG1 switching^48^ and IL-13 can substitute for functions of IL-4^49^, as both cytokines might have been involved in switching to IgG1 in our models. Interestingly, because IL-22 has been recently reported to mediate cardioprotective functions, its detection in vaccine recipients may be an added advantage in maintaining healthy cardiac tissue^50^. Additionally, by evaluating anti-viral cytokines, we noted that Mt 10 virus led to elevated levels of chemokines (IP-10, KC, MCP-1), as well as GM-CSF. Reports indicate that IP-10 enhances immune response against influenza infections^51^, and KC has been reported to be elevated for protection during Theiler’s murine encephalomyelitis virus infection^52^. Similarly, MCP-1 can promote broad-spectrum anti-viral effects in Dengue virus and Japanese encephalitis virus infections^53^, and GM-CSF can attenuate viral replication^54^ and also act as an adjuvant in therapeutic vaccines^55^. Thus, a combination of chemokines and T cell derived cytokines might contribute towards an anti-viral defense mechanism to Mt 10 virus. However, failure to detect classic anti-viral innate cytokines (IFN-α/β) might be because we analyzed these cytokines after day 4 post-vaccination. Their presence may become evident at earlier time points, which we have not examined in this study. Finally, by investigating the safety aspects of Mt 10 virus, we noted no generation of autoimmune response, which otherwise would be expected in wt CVB3 infection, as we have previously demonstrated^10,19^. Lack of increase in the cardiac injury marker cTnI in vaccine recipients lent further support to the proposition that the Mt 10 virus could be safely used as a vaccine virus.

In summary, we have described a novel attenuated strain of CVB3 called Mt 10 virus that serves as a vaccine candidate in the prevention of both myocarditis and pancreatitis in the mouse model of CVB3 infection. The Mt 10 virus induces IgG nABs accompanied with the generation of IFN-γ-producing antigen-specific T cells; vaccine recipients had no detectable viral RNA, indicating that the vaccine responses can effectively prevent infection in challenge studies. Translationally, there is merit in using the Mt 10 virus as a vaccine candidate to prevent CVB3 infection. But it should be noted that six CVB serotypes exist, and more than one serotype can induce similar disease^56^. As such, development of a vaccine for each serotype is challenging, and such an approach may not be viable practically. Thus, we intend to assess whether vaccine responses induced by the Mt 10 virus can also offer cross-protection against multiple serotypes of CVB; indeed, our preliminary data support this possibility (data not shown). Finally, reports indicate that CVB3 has been used to deliver foreign genes of vaccine importance or cytokines in vivo^57–60^. To this end, utility of the Mt 10 virus can be exploited that offer an added advantage in vaccine studies.

## Materials and methods

### Mice

Six-to-eight-week-old male and female A/J mice (H-2^a^) were procured from the Jackson Laboratory (Bar Harbor, ME) and maintained according to the Institutional guidelines of the University of Nebraska-Lincoln, Lincoln, NE, and approval for animal studies was granted by the Institutional Animal Care and Use Committee, UNL (protocol #1904, approved January 2, 2020). The study was carried out in compliance with the ARRIVE guidelines^61^. Infection studies were performed based on biosafety level 2 guidelines. When animals were found to have persistent clinical signs, such as failure to move when physically touched or prodded, or did not eat or drink, they were euthanized using a carbon dioxide chamber as recommended by the Panel on Euthanasia of the American Veterinary Medical Association.

### Peptides and proteins

All peptides (VP1 681–700, RFDLELTFVITSTQQPSTTQ; VP1 721–740, PDKVDSYVWQTST NPSVFWT; RNase 43–56, VNTFVHESLADVQA, and Myhc-α 334–352, DSAFDVLSFTAEEKAGVYK were synthesized by 9-fluorenylmethyloxycarbonyl chemistry (Neopeptide, Cambridge, MA). The purity of peptides was ascertained to be more than 90% via high-performance liquid chromatography, and their identity was confirmed by mass spectroscopy. Ultra-pure water or phosphate-buffered saline (PBS) was used to dissolve the peptides, and multiple aliquots of peptides were stored at  − 20 °C until further use. Full-length CVB3 VP1 (GenScript, Piscataway, NJ) and KLH protein (Sigma-Aldrich, St. Louis, MO) were procured commercially.

### Localization of CVB3 VPs, and analysis of CAR-interacting residues of VP1 771–790

By using the published crystal structure of CVB3 (PDB: 1COV)^16^ , the footprints of binding region between virus and CAR were retrieved to localize the VPs 1 to 4. Potential interactions of amino acid residues within the VP1 771–790 with the host receptor (CAR) were analyzed using the BIOVIA Discovery Studio v 4.0 (Dassault Systèmes, Vélizy-Villacoublay, France).

### Creation of CVB3 mutants and virus recovery studies

CVB3 mutant viruses were generated by site-directed mutagenesis using a CVB3-infectious clone, pBRCVB3, developed previously in our laboratory^11^. The buried amino acid residues within VP1 771–790 were targeted for site-directed mutagenesis. The nucleotides (nts) for amino acid residues at VP1 775, 776, 777, 780, 786, 787, 789 and 790 were replaced with the nts for alanine (A) residues, whereas the nts for A at VP1 788 were changed to G individually, yielding a total of nine mutant clones. Two additional mutants were derived by combining mutations together at residues 775 to 777 (Mt 5) for A, or residues 786 to 790 (Mt 11) for A or G as described above. The PCR products containing the mutations were cloned into the full-length infectious cDNA clone^11^, from which in vitro RNA transcripts were transcribed using T7 RNA polymerase at 37 °C, as recommended (Promega, Madison, WI)^11^. Vero cells were grown to 80% confluency, then trypsinized and washed twice with 1 × PBS by centrifugation at 400xg for 6 min at room temperature (RT). After cells were resuspended in electroporation buffer (BioRad, Hercules, CA) to a cell density of 10 × 10^6^ cells/ml, the in vitro transcribed viral RNA (6 µg) was transferred to a 0.2 cm electroporation cuvette (BioRad), and 200 µl of cell suspension was added. The mixture was subjected to electroporation using Gene Pulser Xcell Electroporation System at 160 V, according to the manufacturer’s recommendations (BioRad). The electroporated cells were gently aspirated and transferred to 6-well plates containing 2 ml of fresh EMEM/10% fetal bovine serum (FBS) prewarmed to 37 °C. Medium was removed after 16 h, cells were washed with 1 × PBS, and replaced with 2 ml of fresh EMEM/2% FBS, then incubated up to 5 days. As the CPE became evident, supernatants containing mutant virus were harvested, passaged, titrated, and stored in aliquots at -80 °C till further use.

### Immunofluorescence assay

We used CVB3 anti-serum (American Type Culture Collection [ATCC], Manassas, VA) to verify the infectivity of viruses derived from mutant clones, allowing us to detect cells expressing viral proteins based on immunofluorescence^62^. Briefly, Vero cells (0.25 × 10^6^ cells/ml/well) were plated on to sterile coverslips in 12-well plates and incubated at 37 °C. After adhesion of Vero cells, the monolayers were washed with 1 × PBS and infected with wt CVB3 (Nancy strain, ATCC), pBRCVB3^11^ (positive controls), and mutant viruses at an MOI of 0.5 for 1.5 h. After adsorption, inoculum was aspirated and replaced with EMEM supplemented with 2% FBS (ATCC). Cells grown in medium alone were used as negative controls. Cells were incubated for 12 h at 37 °C, then washed and fixed in methanol/acetone (1:1) for 40 min at RT. They were then washed three times with 1 × PBS and incubated with anti-CVB3 serum (1:200) in PBS containing 2.5% bovine serum albumin for one hour at RT. After five washings with PBS/Tween 20 (PBST, 0.05%), cells were incubated with fluorescein isothiocyanate (FITC)-conjugated secondary rabbit anti-horse IgG (1:1000; Sigma-Aldrich, St. Louis, MO) for one hour at RT. Finally, coverslips containing the cells were washed, mounted, and visualized under a Nikon A1-Eclipse 90i confocal microscope system (Nikon Instruments Inc-Americas, Melville, NY). Images were acquired sequentially at an excitation/emission wavelength of 561.5 nm/553–618 nm laser set up for pseudo-colored green channel at 60 × magnification.

### Virus propagation and titration

Vero cells (ATCC) grown to 80–90% confluency were infected with wt CVB3, pBRCVB3, and mutant viruses as described previously^11^. After the CPE was confirmed, culture supernatants containing virus were harvested, and viral stocks were stored at  − 80 °C in aliquots until further use. The viruses were titrated, and tissue culture infective dose 50 (TCID)_50_ values were determined according to the Spearman-Karber method^63^. To compare the growth characteristics of the wt CVB3, pBRCVB3, and mutant viruses, Vero cells grown in 12-well plates were infected in triplicates at MOIs 0.1 and 3.0. Culture supernatants were collected at various days post-infection and virus titers were determined as above.

### Animal infection studies

For infection studies, the virus stock was diluted in 1 × PBS to contain 10,000 TCID_50_/200 µl, and the inocula were administered into A/J mice intraperitoneally (i.p). The control (uninfected) mice received only 1 × PBS. Mice were housed 2 to 3 per cage in filter-top cages assembled with closed air-circulation. Cages containing the chow diet and waterers were changed every 3 days until the end of the experiment. The animals had ad libitum access to food and water during the entire study period. Animals were inspected twice daily, and body weights were recorded every 1 to 2 days. An alternative food and fluid source, trans gel diet (ClearH_2_O, Portland, ME), was placed on the cage floor as needed. Hearts and pancreata were collected from animals that died naturally. At termination (day 21 post-infection), animals were euthanized, and hearts, and pancreata were collected.

### Histopathology

Hearts and pancreata were fixed by immersion in 10% phosphate-buffered formalin. Each tissue was sliced into three representative 5 µm cross-sections and stained with hematoxylin and eosin (H and E)^64^. The sections were blinded to treatment and examined by board-certified pathologist, Dr. David Steffen. Pathology scores were generated by enumerating the inflammatory foci, necrosis, mineralization, and fibrosis. Individual scores were used to compare the qualitative nature of the lesions. Total scores represented total foci of pathologic change across the three sections of heart. Multiple changes present in a single focus were counted as 1 in the total count^10,11,65^. The severity of pancreatic change was estimated as percent of tissue section involvement from one random section of pancreas. The nature of pancreatic lesions was noted as atrophy, inflammation, mineralization, and necrosis or a combination of these^10,65^.

### Challenge studies

Groups of mice were administered Mt 10 virus (0.5 × 10^6^ TCID_50_/200 µl in 1 × PBS, i.p.) on day 0. After 14 days, animals were injected with saline or wt CVB3 (10,000 TCID_50_/200 µl in 1 × PBS, i.p.), and body weights were taken every two days thereafter. Hearts and pancreata were collected from animals that died naturally. At termination (21 days post-challenge), animals were euthanized, and serum, spleens, lymph nodes, hearts, and pancreata were collected for further analysis.

### Virus neutralization assay

A virus neutralization test was performed using the sera obtained from different groups. Vero cells were plated at 0.25 × 10^6^ cells/ml in 96 well plates to obtain 90–100% confluency for infection. Serum samples were heat-inactivated at 56 °C for 30 min prior to testing, and twofold serial dilutions were then made (1:10 to 1:20,480). An equal volume of wt CVB3 suspension containing 100 TCID_50_/ml was incubated with serially diluted sera at 37 °C for 1 h in a humidified chamber with 5% CO_2_. After incubation, 100µl of the mixture of each dilution was added in quadruplets to plates containing monolayers of cells and incubated at 37 °C. After four days, plates were observed for CPE, and the highest serum dilution that showed protection from CPE was considered to be the neutralization titer.

### Determination of CVB3-reactive antibodies

Serum samples collected from groups of mice that received Mt 10 virus or saline on days 14 and 35 were analyzed for total Ig, IgG1, IgG2a, IgG2b, IgG3, IgM, IgA, and IgE as described previously^66,67^. In brief, 96-well polystyrene microtiter plates were coated with or without CVB3 VP1 or an irrelevant control (KLH) (5 μg/ml) in 1 × coating buffer (eBioscience, San Diego, CA) and incubated at 4 °C overnight. Plates were washed with 1 × PBS/0.05% Tween-20 and blocked with 1 × PBS/2% BSA/5% normal goat serum for 1.5 h at RT (1:150), and then serum samples were added in duplicates. The plates were incubated at 37 °C for 1 h, and then washed. Horse-radish peroxide (HRP)-labeled goat anti-mouse IgA, IgE, IgM, IgG1, IgG2a, IgG2b, IgG3, and total Ig were then added as secondary antibodies (Southern Biotech, Birmingham, AL). After the plates were incubated at RT for 2 h, 1 × tetramethylbenzidine (TMB) solution was added as a substrate (eBioscience) and reactions were stopped using 1 M phosphoric acid. Plates were read at 450 nm using an automated ELISA reader (BioTek instruments, Winooski, VT), and optical density (OD) values were measured^66,68^.

### MHC-II dextramer/tetramer staining

We recently created MHC class II/IA^k^ tetramers and dextramers to enumerate the frequencies of CD4 T cells specific to VP1 681–700 and VP1 721–740^12^. In this study, we used IA^k^/dextramers for VP1 681–700 and IA^k^/tetramers for VP1 721–740, which were available at the time. Briefly, single cell suspensions of lymphocytes were obtained from mice injected with Mt 10 virus or saline. Cells were stimulated with VP1 681–700 and VP1 721–740 (20 µg/ml) for 2 days, and viable cells were maintained in medium containing IL-2^12^. During the 7 to 10 days post-stimulation, cells were stained with the VP1 dextramers and tetramers described above, as well as their corresponding control dextramers and tetramers (RNase 43–56), followed by anti-CD4 (GK1.5, BioLegend, San Diego, CA) and 7-aminoactinomycin D (7-AAD, Invitrogen, Carlsbad, CA)^69–71^. After acquiring the cells by flow cytometry (FACSCalibur, BD Biosciences, CA), percentages of dextramer or tetramer positive cells were determined in the live (7-AAD^¯^) CD4^+^ subset using FlowJo software [v 7.6.5] (Tree Star, Ashland, OR)^12^.

### Cytokine analysis

Supernatants obtained from lymphocyte cultures prepared from animals injected with saline or Mt 10 virus were stimulated with or without VP1 681–700 and VP1 721–740 (20 μg/ml) on day 3 post-stimulation. Cytokine analysis was performed using the LEGENDplex Murine Th cytokine Panel (12-plex; BioLegend). Cytokine analysis included a panel of IL-2, IFN-γ, IL-4, IL-5, IL-6, IL-9, IL-10, IL-13, IL-17A, IL-17F, IL-22, and TNF-α. Similarly, serum samples obtained from saline and Mt 10 virus groups were evaluated for a panel of anti-viral cytokines and chemokines using LEGENDplex Murine anti-viral kit (13-plex; BioLegend). These included IFN-γ, KC, TNF-α, MCP-1, IL-12p70, RANTES, IL-1β, IP-10, GM-CSF, IL-10, IFN-β, IFN-α, and IL-6. Standard curves were obtained by serially diluting the lyophilized mouse cytokine standard mix provided in the kit. Briefly, capture bead/cytokine antibody conjugates were first prepared, and the mixtures were added to a tube containing diluted standards or test samples, followed by addition of detection antibodies and streptavidin–phycoerythrin reagents. After acquisition by flow cytometry, cytokine concentrations were determined using the LEGENDplex data analysis software suite (BioLegend).

### Evaluation of Myhc-reactive T cell responses

Lymphocytes obtained from groups of animals that received wt CVB3 or Mt 10 virus and Mt 10 virus/wt CVB3 were used to assess Myhc-α 334–352-reactive T cell responses based on tritiated ^3^[H] thymidine-incorporation assay^66,72,73^. In brief, cells were suspended in RPMI containing 10% FBS, 1 mM sodium pyruvate, 4 mM l-glutamine, 1 × each of non-essential amino acids and vitamin mixture, and 100 U/ml penicillin–streptomycin (Lonza, Basel, Switzerland). Cells were stimulated with Myhc-α 334–352 (50 µg/ml) or RNase 43–56 (50 µg/ml) for 2 days at a density of 5 × 10^6^ cells/ml in triplicates, whereas cells cultured with no peptides were used as medium controls. After pulsing with ^3^[H] thymidine (1µ curie/well; Moravek Inc., Brea, CA) for 16 h, proliferative responses were measured as counts per minute (cpm) using a Wallac liquid scintillation counter (Perkin Elmer, Waltham, MA).

### Measurement of serum cTnI

Serum cTnI levels were measured in the sera collected from saline, wt CVB3, Mt 10 virus, and Mt 10 virus/wt CVB3 groups using the high-sensitivity cTnI ELISA kit according to manufacturer’s recommendations (Life Diagnostics Inc., West Chester, PA). Briefly, 100 μl of cTnI HRP conjugate and 100 μl of standards/samples were added to anti-cTnI-coated wells. After mixing, plates were incubated on an orbital shaker for 1 h at RT, followed by addition of 100µL TMB as substrate and an equal volume of stop solution. Plates were read at 450 nm using an automated ELISA reader to obtain the OD values (BioTek instruments). Concentration of cTnI in unknown samples was calculated using the standard curve generated by plotting the absorbance values of the standards versus log_10_ of their concentration.

### RNA isolation and real-time quantitative PCR

For RNA isolation, hearts and pancreata stored at  − 80 °C were used. Approximately 20–30 mg of tissue were transferred to the RLT buffer and homogenized with a FastPrep96 system as recommended (Lysing Matrix D 1.4-mm ceramic beads; MP Biomedicals, Irvine, CA). RNA was isolated using the RNeasy kit (Qiagen, Hilden, Germany), and samples were treated with deoxyribonuclease (DNase) I and quantified using the NanoDrop ND-1000 spectrophotometer (Thermo Fisher Scientific, Waltham, MA). In a single-step reaction, RNA was reverse-transcribed and PCR was performed using the iTaq Universal one-step RT-qPCR kit (BioRad, Hercules, CA). The real-time quantitative PCR analysis included amplifications for CVB3 VP1 (target gene) and glyceraldehyde-3-phosphate dehydrogenase (GAPDH, house-keeping gene) using TaqMan Gene Expression Assays (Applied Biosystems) and the CFX96 Touch Real-time PCR detection system (BioRad). Expression of CVB3 VP1 was normalized to GAPDH using the 2^−(∆∆*C*t)^ method.

### Statistical analysis

Statistical analyses were performed using GraphPad Prism software v8.0 (GraphPad Software, Inc. La Jolla, CA, USA). Data sets pertaining to viral titers, tetramer^+^ and dextramer^+^ cells, antibodies, cytokines, T cell proliferation, cTnI, and qPCR were analyzed by unpaired student’s t-test, Mann–Whitney test, or Kruskal–Wallis test for pairwise comparisons between the groups. Two-way ANOVA with Sidak’s post-test was used to compare the body weight changes. Log-rank test with Bonferroni correction was used to analyze statistical significance of the survival curves. Barnard’s exact test was used to analyze the histological parameters^74^. Graphs were prepared by GraphPad Prism software v8.0.

## Supplementary Information

Supplementary Information.

## References

[1] Zaoutis T, Klein JD (1998). Enterovirus infections. Pediatr. Rev. Am. Acad. Pediatr..

[2] Rhoades RE, Tabor-Godwin JM, Tsueng G, Feuer R (2011). Enterovirus infections of the central nervous system. Virology.

[3] Cihakova D, Rose NR (2008). Pathogenesis of myocarditis and dilated cardiomyopathy. Adv. Immunol..

[4] Huber S, Ramsingh AI (2004). Coxsackievirus-induced pancreatitis. Viral Immunol..

[5] Wong AH, Lau CS, Cheng PK, Ng AY, Lim WW (2011). Coxsackievirus B3-associated aseptic meningitis: an emerging infection in Hong Kong. J. Med. Virol..

[6] Archard LC (1991). Molecular probes for detection of persisting enterovirus infection of human heart and their prognostic value. Eur. Heart J..

[7] Kuhl U (2005). High prevalence of viral genomes and multiple viral infections in the myocardium of adults with "idiopathic" left ventricular dysfunction. Circulation.

[8] Martino, T., Liu, P. & Sole, M. J. *Enteroviral myocarditis and dialted cardiomyopathy: a review of clinical and experimental studies. In Human Enterovirus infections.*, 291–351 (ASM, 1995).

[9] Chapman NM, Kim KS (2008). Persistent coxsackievirus infection: enterovirus persistence in chronic myocarditis and dilated cardiomyopathy. Curr. Top Microbiol. Immunol..

[10] Gangaplara A (2012). Coxsackievirus B3 infection leads to the generation of cardiac myosin heavy chain-alpha-reactive CD4 T cells in A/J mice. Clin. Immunol..

[11] Massilamany C (2015). Mutations in the 5' NTR and the non-structural protein 3A of the coxsackievirus B3 selectively attenuate myocarditogenicity. PLoS ONE.

[12] Lasrado N (2020). Identification of immunogenic epitopes that permit the detection of antigen-specific T cell responses in multiple serotypes of group B coxsackievirus infections. Viruses.

[13] Lewis GK, Feng CP (1992). Intrinsic immunogenicity of an internal VP1 T-B epitope pair of type 1 poliovirus. Mol. Immunol..

[14] Senkowski A, Shim B, Roos RP (1995). The effect of Theiler's murine encephalomyelitis virus (TMEV) VP1 carboxyl region on the virus-induced central nervous system disease. J. Neurovirol..

[15] Zhang Y (2011). Natural type 3/type 2 intertypic vaccine-related poliovirus recombinants with the first crossover sites within the VP1 capsid coding region. PLoS ONE.

[16] Muckelbauer JK (1995). Structure determination of coxsackievirus B3 to 3.5 A resolution. Acta Crystallogr. D Biol. Crystallogr..

[17] He Y (2001). Interaction of coxsackievirus B3 with the full length coxsackievirus-adenovirus receptor. Nat. Struct. Biol..

[18] Schultz CL, Coffman RL (1991). Control of isotype switching by T cells and cytokines. Curr. Opin. Immunol..

[19] Basavalingappa RH (2020). Viral myocarditis involves the generation of autoreactive T cells with multiple antigen specificities that localize in lymphoid and non-lymphoid organs in the mouse model of CVB3 infection. Mol. Immunol..

[20] Donermeyer DL, Beisel KW, Allen PM, Smith SC (1995). Myocarditis-inducing epitope of myosin binds constitutively and stably to I-Ak on antigen-presenting cells in the heart. J. Exp. Med..

[21] Adams JE (1993). Cardiac troponin I. A marker with high specificity for cardiac injury. Circulation.

[22] Tschope C (2020). Myocarditis and inflammatory cardiomyopathy: current evidence and future directions. Nat. Rev. Cardiol..

[23] Lasrado N, Reddy J (2020). An overview of the immune mechanisms of viral myocarditis. Rev. Med. Virol..

[24] Lasrado N, Yalaka B, Reddy J (2020). Triggers of inflammatory heart disease. Front. Cell Dev. Biol..

[25] Flynn CT, Kimura T, Frimpong-Boateng K, Harkins S, Whitton JL (2017). Immunological and pathological consequences of coxsackievirus RNA persistence in the heart. Virology.

[26] Seo I (2007). Mutation variants generated from nonvirulent coxsackievirus B3 acquire virulence phenotypes by active virus replication. Intervirology.

[27] Zhang H (1997). Coxsackievirus B3-induced myocarditis. Characterization of stable attenuated variants that protect against infection with the cardiovirulent wild-type strain. Am. J. Pathol..

[28] Hunziker IP, Harkins S, Feuer R, Cornell CT, Whitton JL (2004). Generation and analysis of an RNA vaccine that protects against coxsackievirus B3 challenge. Virology.

[29] M'Hadheb-Gharbi MB, Paulous S, Aouni M, Kean KM, Gharbi J (2007). The substitution U475 –> C with Sabin3-like mutation within the IRES attenuate Coxsackievirus B3 cardiovirulence. Mol. Biotechnol..

[30] Qi X, Lu Q, Hu J, Xiong S (2019). Spontaneous C-cleavage of a truncated intein as fusion tag to produce tag-free VP1 inclusion body nanoparticle vaccine against CVB3-induced viral myocarditis by the oral route. Microb. Cell Fact..

[31] Kim JY (2005). Immunogenicity of a DNA vaccine for coxsackievirus B3 in mice: protective effects of capsid proteins against viral challenge. Vaccine.

[32] Wu F, Fan X, Yue Y, Xiong S, Dong C (2014). A vesicular stomatitis virus-based mucosal vaccine promotes dendritic cell maturation and elicits preferable immune response against coxsackievirus B3 induced viral myocarditis. Vaccine.

[33] Zhang L (2012). Vaccination with coxsackievirus B3 virus-like particles elicits humoral immune response and protects mice against myocarditis. Vaccine.

[34] Park JH (2009). Attenuation of coxsackievirus B3 by VP2 mutation and its application as a vaccine against virus-induced myocarditis and pancreatitis. Vaccine.

[35] Stone VM (2020). A hexavalent Coxsackievirus B vaccine is highly immunogenic and has a strong protective capacity in mice and nonhuman primates. Sci. Adv..

[36] Bergelson JM (1997). Isolation of a common receptor for Coxsackie B viruses and adenoviruses 2 and 5. Science.

[37] Massilamany C, Gangaplara A, Reddy J (2014). Intricacies of cardiac damage in coxsackievirus B3 infection: implications for therapy. Int. J. Cardiol..

[38] Marchant D, Si X, Luo H, McManus B, Yang D (2008). The impact of CVB3 infection on host cell biology. Curr. Top Microbiol. Immunol..

[39] Shafren DR, Williams DT, Barry RD (1997). A decay-accelerating factor-binding strain of coxsackievirus B3 requires the coxsackievirus-adenovirus receptor protein to mediate lytic infection of rhabdomyosarcoma cells. J. Virol..

[40] Steel J (2009). Live attenuated influenza viruses containing NS1 truncations as vaccine candidates against H5N1 highly pathogenic avian influenza. J. Virol..

[41] Whitehead SS (2003). A live, attenuated dengue virus type 1 vaccine candidate with a 30-nucleotide deletion in the 3' untranslated region is highly attenuated and immunogenic in monkeys. J. Virol..

[42] Yee PTI (2019). Development of live attenuated Enterovirus 71 vaccine strains that confer protection against lethal challenge in mice. Sci. Rep..

[43] Cheng X (2013). Evaluation of the humoral and cellular immune responses elicited by the live attenuated and inactivated influenza vaccines and their roles in heterologous protection in ferrets. J. Infect. Dis..

[44] Snapper CM, Peschel C, Paul WE (1988). IFN-gamma stimulates IgG2a secretion by murine B cells stimulated with bacterial lipopolysaccharide. J. Immunol..

[45] Severinson E, Fernandez C, Stavnezer J (1990). Induction of germ-line immunoglobulin heavy chain transcripts by mitogens and interleukins prior to switch recombination. Eur. J. Immunol..

[46] Deenick EK, Hasbold J, Hodgkin PD (2005). Decision criteria for resolving isotype switching conflicts by B cells. Eur. J. Immunol..

[47] McIntyre TM (1993). Transforming growth factor beta 1 selectivity stimulates immunoglobulin G2b secretion by lipopolysaccharide-activated murine B cells. J. Exp. Med..

[48] Snapper CM, Finkelman FD, Paul WE (1988). Regulation of IgG1 and IgE production by interleukin 4. Immunol. Rev..

[49] Chomarat P, Banchereau J (1998). Interleukin-4 and interleukin-13: their similarities and discrepancies. Int. Rev. Immunol..

[50] Guo Y (2014). IL-22-producing Th22 cells play a protective role in CVB3-induced chronic myocarditis and dilated cardiomyopathy by inhibiting myocardial fibrosis. Virol. J..

[51] Chan MC (2005). Proinflammatory cytokine responses induced by influenza A (H5N1) viruses in primary human alveolar and bronchial epithelial cells. Respir. Res..

[52] Kang MH, Jin YH, Kim BS (2018). Effects of keratinocyte-derived cytokine (CXCL-1) on the development of Theiler's virus-induced demyelinating disease. Front. Cell Infect. Microbiol..

[53] Lin RJ (2013). MCPIP1 ribonuclease exhibits broad-spectrum antiviral effects through viral RNA binding and degradation. Nucl. Acids Res..

[54] Bukreyev A, Belyakov IM, Berzofsky JA, Murphy BR, Collins PL (2001). Granulocyte-macrophage colony-stimulating factor expressed by recombinant respiratory syncytial virus attenuates viral replication and increases the level of pulmonary antigen-presenting cells. J. Virol..

[55] Zhao W, Zhao G, Wang B (2018). Revisiting GM-CSF as an adjuvant for therapeutic vaccines. Cell Mol. Immunol..

[56] M.S. Oberste, M. A. P. Coxsackieviruses. *Encyclopedia of Virology*, 3rd edn., 580–587, 10.1016/B978-012374410-4.00372-1 (2008).

[57] Chapman NM (2000). Coxsackievirus expression of the murine secretory protein interleukin-4 induces increased synthesis of immunoglobulin G1 in mice. J. Virol..

[58] Henke A, Jarasch N, Martin U, Zell R, Wutzler P (2008). Characterization of the protective capability of a recombinant coxsackievirus B3 variant expressing interferon-gamma. Viral. Immunol..

[59] Henke A, Zell R, Ehrlich G, Stelzner A (2001). Expression of immunoregulatory cytokines by recombinant coxsackievirus B3 variants confers protection against virus-caused myocarditis. J. Virol..

[60] Kim DS, Cho YJ, Kim BG, Lee SH, Nam JH (2010). Systematic analysis of attenuated Coxsackievirus expressing a foreign gene as a viral vaccine vector. Vaccine.

[61] Percie du Sert N (2020). The ARRIVE guidelines 2.0: updated guidelines for reporting animal research. PLOS Biol..

[62] Pan J (2011). Single amino acid changes in the virus capsid permit coxsackievirus B3 to bind decay-accelerating factor. J. Virol..

[63] Dougherty, R. & Harris, R. Techniques in experimental virology. **169** (1964).

[64] Afanasyeva M (2001). Experimental autoimmune myocarditis in A/J mice is an interleukin-4-dependent disease with a Th2 phenotype. Am. J. Pathol..

[65] Massilamany C, Gangaplara A, Steffen D, Reddy J (2011). Identification of novel mimicry epitopes for cardiac myosin heavy chain-alpha that induce autoimmune myocarditis in A/J mice. Cell Immunol..

[66] Basavalingappa RH (2016). Identification of an epitope from adenine nucleotide translocator 1 that induces inflammation in heart in A/J mice. Am. J. Pathol..

[67] Krishnan B (2018). Epitope mapping of SERCA2a identifies an antigenic determinant that induces mainly atrial myocarditis in A/J mice. J. Immunol..

[68] Storck S (2005). Normal immune system development in mice lacking the Deltex-1 RING finger domain. Mol. Cell. Biol..

[69] Massilamany C, Steffen D, Reddy J (2010). An epitope from Acanthamoeba castellanii that cross-react with proteolipid protein 139–151-reactive T cells induces autoimmune encephalomyelitis in SJL mice. J. Neuroimmunol..

[70] Reddy J (2003). Detection of autoreactive myelin proteolipid protein 139–151-specific T cells by using MHC II (IAs) tetramers. J. Immunol..

[71] Massilamany C, Gangaplara A, Chapman N, Rose N, Reddy J (2011). Detection of cardiac myosin heavy chain-alpha-specific CD4 cells by using MHC class II/IA(k) tetramers in A/J mice. J. Immunol. Methods.

[72] Krishnan B (2017). Branched chain α-ketoacid dehydrogenase kinase 111–130, a T cell epitope that induces both autoimmune myocarditis and hepatitis in A/J mice. Immun. Inflamm. Disease.

[73] Massilamany C, Gangaplara A, Steffen D, Reddy J (2011). Identification of novel mimicry epitopes for cardiac myosin heavy chain-α that induce autoimmune myocarditis in A/J mice. Cell. Immunol..

[74] Barnard GA (1945). A new test for 2 × 2 tables. Nature.

