# Supplementary material for: Attenuated strain of CVB3 with a mutation in the CAR-interacting region protects against both myocarditis and pancreatitis
Source: Sci Rep. 2021 Jun 14;11:12432. doi: 10.1038/s41598-021-90434-w (PMC8203608; doi:10.1038/s41598-021-90434-w)
Supplement: Supplementary file 1 — Supplementary Information. [file 41598_2021_90434_MOESM1_ESM.pdf]

Attenuated strain of CVB3 with a mutation in the CAR-interacting region protects against both myocarditis and pancreatitis

Ninaad Lasrado[a], Arunakumar Gangaplara[a,1], Chandirasegaran Massilamany[a,2], Rajkumar Arumugam[a], Allison Shelbourn[a], Mahima T. Rasquinha[a], Rakesh H. Basavalingappa[a,3], Gustavo Delhon[a], Shi-Hua Xiang[a,b], Asit K. Pattnaik[a,b], David Steffen[a], and Jay Reddy[a*]

[a]School of Veterinary Medicine and Biomedical Sciences, University of Nebraska-Lincoln, Lincoln, NE; [b]Nebraska Center for Virology, University of Nebraska-Lincoln, Lincoln, NE;

Present address:

[1]Laboratory of Early Sickle Mortality Prevention, Cellular and Molecular Therapeutics Branch, National Heart, Lung, and Blood Institute, National Institutes of Health, Bethesda, MD

[2]CRISPR Therapeutics, Cambridge, MA

[3]Aclaris Therapeutics, Inc., St. Louis, MO

Running title: Mutations in the CVB3 genome affecting disease phenotype

***Corresponding author:**
Jay Reddy MVSc., PhD
Room 202, Bldg. VBS
School of Veterinary Medicine and Biomedical Sciences
University of Nebraska-Lincoln
Lincoln, NE 68583
Phone: (402) 472 8541
Fax: (402) 472 9690
E-mail: nreddy2@unl.edu

Table S1: Amino acid and nucleotide codons of VP1 771-790

Amino acid position	Amino acids	Codons
771	Arginine (R)	AGG
772	Asparagine (N)	AAC
773	Glycine (G)	GGA
774	Valine (V)	GTT
775	Tyrosine (Y)	TAC
776	Glycine (G)	GGC
777	Isoleucine (I)	ATC
778	Asparagine (N)	AAC
779	Threonine (T)	ACG
780	Leucine (L)	CTA
781	Asparagine (N)	AAC
782	Asparagine (N)	AAC
783	**Methionine (M)**	**ATG**
784	**Glycine (G)**	**GGC**
785	**Threonine (T)**	**ACG**
786	Leucine (L)	CTA
787	Tyrosine (Y)	TAT
788	Alanine (A)	GCA
789	Arginine (R)	AGA
790	Histidine (H)	CAT

Note: Bold residues (M, G and T) are partially buried, but they interact with CAR. Underlined residues (Y, G, I, L, A, L, Y, R, and H) are buried, but do not interact with CAR.

Table S2: List of primers used for the creation of CVB3 mutant viruses

Sl No.	Primer name	Sequence
1	SPEI-P2 Reverse Primer	5' ggagtcttgacccactagtgattctttc 3'
2	XHOI-P3 Forward Primer	5' accagggtactcgagtgtttttag 3'
3	Mt 1 Forward Primer	5' ccaggaacggagttgccggcatcaa 3'
4	Mt 2 Forward Primer	5' ccaggaacggagtttacgccatcaacac 3'
5	Mt 3 Forward Primer	5' ttacggcgccaacacgctaaac 3'
6	Mt 4 Forward Primer	5' atcaacacggcaaacaacatg 3'
7	Mt 5 Forward Primer	5' ccaggaacggagttgccgccgccaacacg 3'
8	Mt 6 Forward Primer	5' aaacaacatgggcacggcatatgcaag 3'
9	Mt 7 Forward Primer	5' gggcacgctagctgcaagacatg 3'
10	Mt 8 Forward Primer	5' gggcacgctatatggaagacatgt 3'
11	Mt 9 Forward Primer	5' gggcacgctatatgcagcacatgtcaac 3'
12	Mt 10 Forward Primer	5' atatgcaagagctgtcaacgctgg 3'
13	Mt 11 Forward Primer	5' gggcacggcagctggagcagctgtcaacgc 3'
14	SPE-CVB Forward Primer	5' ataccaatcccatgtgcttttag 3'
15	SPE-CVB Reverse Primer	5' aaccgataagggctagtgtggcag 3'

Table S3: The list of mutations introduced within VP1 771-790 in the CVB3 genome

**Amino acid position**	**Residue/s to be mutated to Alanine/Glycine**	**Original codon**	**Mutated codon**
VP1 775	Y to A (Tyrosine to Alanine)	**TAC**	**GCC**
VP1 776	G to A (Glycine to Alanine)	**GGC**	**GCC**
VP1 777	I to A (Isoleucine to Alanine)	**ATC**	**GCC**
VP1 780	L to A (Leucine to Alanine)	**CTA**	**GCA**
VP1 775-777	YGI to AAA (Tyrosine, Glycine and Isoleucine to Alanine, Alanine and Alanine)	**TACGGCATC**	**GCCGCCGCC**
VP1 786	L to A (Leucine to Alanine)	**CTA**	**GCA**
VP1 787	Y to A (Tyrosine to Alanine)	**TAT**	**GCT**
VP1 788	A to G (Alanine to Glycine)	**GCA**	**GGA**
VP1 789	R to A (Arginine to Alanine)	**AGA**	**GCA**
VP1 790	H to A (Histidine to Alanine)	**CAT**	**GCT**
VP1 786-790	LYARH to AAGAA (Leucine, Tyrosine, Alanine, Arginine and Histidine to Alanine, Alanine, Glycine, Alanine and Alanine)	**CTATATGCAAGACAT**	**GCAGCTGGAGCAGCT**

Table S4: List of original and mutated nucleotide sequences of VP1 771-790 for the derivation of infectious mutant viruses

Mutations	Sequences (original, top; and mutated, bottom)
**1.** Y to A (775)	AGGAACGGAGTTTACGGCATCAACACGCTAAACAACATGGGCACGCTATATGCAAGACAT<br>AGGAACGGAGTT**GC**CGGCATCAACACGCTAAACAACATGGGCACGCTATATGCAAGACAT
**2.** G to A (776)	AGGAACGGAGTTTACGGCATCAACACGCTAAACAACATGGGCACGCTATATGCAAGACAT<br>AGGAACGGAGTTTACG**C**CATCAACACGCTAAACAACATGGGCACGCTATATGCAAGACAT
**3.** I to A (777)	AGGAACGGAGTTTACGGCATCAACACGCTAAACAACATGGGCACGCTATATGCAAGACAT<br>AGGAACGGAGTTTACGGC**GC**CAACACGCTAAACAACATGGGCACGCTATATGCAAGACAT
**4.** L to A (780)	AGGAACGGAGTTTACGGCATCAACACGCTAAACAACATGGGCACGCTATATGCAAGACAT<br>AGGAACGGAGTTTACGGCATCAACACG**GC**AAACAACATGGGCACGCTATATGCAAGACAT
**5.** YGI to AAA (775-777)	AGGAACGGAGTTTACGGCATCAACACGCTAAACAACATGGGCACGCTATATGCAAGACAT<br>AGGAACGGAGTT**GC**CG**C**C**GC**CAACACGCTAAACAACATGGGCACGCTATATGCAAGACAT
**6.** L to A (786)	AGGAACGGAGTTTACGGCATCAACACGCTAAACAACATGGGCACGCTATATGCAAGACAT<br>AGGAACGGAGTTTACGGCATCAACACGCTAAACAACATGGGCACG**GC**ATATGCAAGACAT
**7.** Y to A (787)	AGGAACGGAGTTTACGGCATCAACACGCTAAACAACATGGGCACGCTATATGCAAGACAT<br>AGGAACGGAGTTTACGGCATCAACACGCTAAACAACATGGGCACGCTA**GC**TGCAAGACAT
**8.** A to G (788)	AGGAACGGAGTTTACGGCATCAACACGCTAAACAACATGGGCACGCTATATGCAAGACAT<br>AGGAACGGAGTTTACGGCATCAACACGCTAAACAACATGGGCACGCTATATG**G**AAGACAT
**9.** R to A (789)	AGGAACGGAGTTTACGGCATCAACACGCTAAACAACATGGGCACGCTATATGCAAGACAT<br>AGGAACGGAGTTTACGGCATCAACACGCTAAACAACATGGGCACGCTATATGCA**GC**ACAT
**10.** H to A (790)	AGGAACGGAGTTTACGGCATCAACACGCTAAACAACATGGGCACGCTATATGCAAGACAT<br>AGGAACGGAGTTTACGGCATCAACACGCTAAACAACATGGGCACGCTATATGCAAGA**GC**T
**11.** LYARH to AAGAA (786-790)	AGGAACGGAGTTTACGGCATCAACACGCTAAACAACATGGGCACGCTATATGCAAGACAT<br>AGGAACGGAGTTTACGGCATCAACACGCTAAACAACATGGGCACG**GC**A**GC**TG**G**A**GC**A**GC**T
